# From diversity to dominance: how salt and CO₂ shape LAB-dominated ecosystems in vegetable fermentations

**DOI:** 10.1128/spectrum.03578-25

**Published:** 2026-06-30

**Authors:** Tom Eilers, Tim Van Rillaer, Stijn Wittouck, Ines Tuyaerts, Katrien Michiels, Maline Victor, Thies Gehrmann, Peter A. Bron, Wannes Van Beeck, Sarah Lebeer

**Affiliations:** 1 Department of Bioscience Engineering, Lab of Applied Microbiology and Biotechnology, University of Antwerp 692679, Antwerp, Belgium; 2 U-MaMi Centre of Excellence, University of Antwerp 26660, Antwerp, Belgium; University of Mississippi, University, Mississippi, USA; University of Torino, Torino, Italy

**Keywords:** microbial succession, food fermentation, lactic acid bacteria

## Abstract

Research on microbial ecosystems is often challenging due to the high diversity of microbial taxa present and the complexity of controlling environmental variables. Fermented foods offer simpler and more reproducible model ecosystems in which both community composition and environmental factors can be more precisely controlled and manipulated. In this study, we focused on fermented vegetables, which are typically dominated by lactic acid bacteria (LAB). However, it remains unclear why LAB consistently drive the spontaneous fermentation of vegetables and how factors such as vegetable substrates, salt addition, and carbon dioxide levels shape microbial community dynamics. We characterized the temporal microbial succession in standardized spontaneous fermentations of 11 different vegetables (including beetroot, bell pepper, cabbage, carrot, cucumber, fennel, green asparagus, leek, parsnip, sunroot, and tomato), revealing a robust and recurrent dominance of *Leuconostoc* and other LAB across substrates. Additionally, we investigated the impact of varying salt concentrations and found that lower salt levels delayed the establishment of the typically LAB-dominated community*,* while promoting a higher abundance of *Weissella* and multiple *Enterobacterales* taxa. Notably, these salt reduction-induced effects were mitigated by CO_2_ injection, which reduced *Enterobacterales* levels and increased the overall abundance of *Lactobacillales*. Together, these findings demonstrate how targeted manipulation of environmental parameters, such as salinity and gas composition, can be used to uncover ecological principles governing microbial succession and community assembly in reproducible fermentation-based model ecosystems.

**IMPORTANCE:**

Understanding the ecological principles that shape microbial community assembly is essential for advancing our knowledge of microbial ecosystems. Fermented vegetables, which are increasingly popular among the general population, provide a tractable and reproducible model system to study microbial succession. By systematically manipulating variables such as vegetable substrate, salinity, and gas composition, we identified the effects of these factors on microbial dynamics throughout the fermentation. These insights not only enhance our understanding of the microbial ecology of these man-made food systems but also suggest directions for novel strategies to optimize fermentation processes for the production of faster, safer, and more flavorful foods.

## INTRODUCTION

Research on microbial ecosystems is often challenging due to the large diversity of microbial taxa and the numerous and often unknown external factors that shape ecosystem development. To better understand the key drivers of microbial dynamics and interactions, simpler, manipulable, and reproducible model systems are needed. Fermented foods offer such natural yet tractable ecosystems, in which external parameters can be systematically varied (1). Their adaptability allows researchers to modulate conditions such as substrate type, salinity, CO₂ levels, temperature, and fermentation duration. This type of mechanistic ecological research not only advances our understanding of microbial community assembly but also supports the development of practical strategies for microbiome engineering in applied contexts.

In recent years, fermented food ecosystems have gained renewed popularity in society, driven by the growing production and consumption of artisanal and industrial fermented products. This resurgence in Western culture is fueled by their desirable organoleptic properties, their potential to promote sustainability by reducing food waste, and increasing evidence of their health benefits (2–6). Fermented foods encompass distinct food products, but here, we adhere to the scientific definition that fermented foods are “foods made through desired microbial growth and enzymatic conversions of food components” (7). It is estimated that over 5,000 different fermented foods exist globally, which can be divided based on the substrate fermented (8). In addition to cow milk, vegetables are among the most important substrates of fermented foods (9).

Vegetable fermentations typically begin with chopping or crushing the vegetables, adding salt, and excluding air by sealing the mixture in airtight jars to create anaerobic or low-oxygen conditions. These conditions selectively stimulate the growth of a desired subset of the native microbial community, primarily lactic acid bacteria (LAB) (10). LAB are known for their ability to ferment plant-derived sugars into lactic acid, which lowers pH and inhibits the growth of undesirable microbes, particularly members of the order *Enterobacterales* (11). These plant-associated taxa typically co-occur with LAB at the onset of fermentation and are linked to the production of off-flavors and potentially harmful compounds such as biogenic amines and toxins (12). However, it remains unclear whether *Enterobacterales* should be considered spoilage-associated since many are common plant-associated bacteria and appear during early fermentations without any health implications (12, 13).

Several vegetable fermentations have been extensively studied so far. These include sauerkraut (14, 15), kimchi (16, 17), cucumber (18), olives (19), and fermented carrot juice (12, 20). Across these and other spontaneous plant fermentations, a characteristic succession is observed. Fermentation starts with the microbes residing on the plants, including but not limited to *Enterobacterales* and LAB, which are gradually replaced by LAB with differing tolerance to acidity. First, acid-sensitive (i.e., inhibition below pH of 3.5–4) taxa, including *Leuconostoc*, *Enterococcus*, and *Lactococcus,* dominate, followed by more acid-tolerant LAB such as the *Lactiplantibacillus, Latilactobacillus*, and *Limosilactobacillus* (12, 14, 21–23). Despite this general pattern, it remains uncertain whether similar microbial dynamics occur in the fermentation of all vegetables. Moreover, the ecological mechanisms driving LAB dominance and succession remain poorly understood. Drivers include the starting substrate’s initial microbial community (24) and nutrient composition (25), as well as broader environmental influences, akin the concept of “microbial terroir” in wine fermentation, where microgeographic climate and environmental conditions shape microbial communities (26). In this context, it is still unknown whether the location on the plant, i.e., soil-exposed, nutrient-dense rhizosphere and air-exposed phyllosphere and carposphere affect the microbial dynamics during fermentation.

Other environmental variables can also be modulated to influence the microbial community dynamics that occur during fermentation. Temperature and salt are among the most critical parameters shaping the community structure (27). Traditionally, variable concentrations of salt, typically ranging from 2% to 8%, are added to vegetable fermentations (20, 28, 29), decreasing the water activity (30) and increasing osmotic stress. However, high salt concentrations are negatively perceived by consumers for organoleptic properties (31) and linked to (presumed) negative health effects of the fermented product (30, 32, 33). Recent studies have explored reducing salt concentrations in traditional fermentations, down to 1.4% in kimchi (21), 0.5% in sauerkraut (34), and 0% in cucumbers (35), while monitoring metabolites and pH dynamics. Although these studies only partially examined the microbial communities, they reported distinct shifts in microbial composition and fermentation profiles. A comprehensive investigation into the complete removal of salt and its effects on the full microbial community remains scarce.

Atmospheric conditions, including the presence of carbon dioxide (CO₂), represent another environmental factor influencing microbial community dynamics during fermentation. Carbon dioxide (CO₂) has been shown to inhibit a broad range of spoilage organisms. For instance, in liquid broth experiments where CO_2_ was introduced via gas packaging, concentrations of 2,000 mg/L CO_2_ completely inhibited several Gram-negative bacteria such as *Pseudomonas fluorescens*, *Photobacterium phosphoreum*, *Shewanella putrefaciens*, and *Aeromonas hydrophila*. Gram-positive bacteria such as *Brochothrix thermosphacta* and *Bacillus circulans* were also inhibited at the same concentration, albeit to a lesser extent (36). In contrast, *Latilactobacillus sakei* exhibited only a moderate reduction in growth rate at concentrations up to 2,500 mg/L CO_2_ (36), suggesting that CO_2_ may act as a selective pressure favoring LAB dominance. Interestingly, CO₂ is also naturally produced during vegetable fermentation by heterofermentative LAB such as *Leuconostoc* spp., yet its potential role in shaping microbial succession remains unexplored. Understanding how atmospheric composition influences microbial ecology could open new avenues for steering fermentation dynamics, particularly in low- or no-salt conditions.

To clarify how substrate characteristics, salinity, and atmospheric conditions (in particular CO₂) shape microbial community assembly and LAB dominance in plant-associated fermentation ecosystems, we conducted a systematic investigation across 11 vegetables (Table 1) representing distinct plant regions, including carposphere (e.g., tomato), phyllosphere (e.g., cabbage), and rhizosphere (e.g., carrot and sunroot). All fermentations were set up in a standardized way and were fermented with a minimum of nine biological replicates each. Active microbial communities were analyzed via 16S-rRNA sequencing at the genus level using the updated *Lactobacillaceae* taxonomy (37). The specific effects of salt and CO₂ were examined using carrot juice fermentations as a controlled model system, enabling detailed analysis of their impact on microbial succession and LAB dominance.

**TABLE 1:** Overview of the various vegetables used in this project^*a*^

Vegetable	Latin name	Plant organ	Region of plant	Replicates	Start pH
Beetroot	*Beta vulgaris v*ar. Ruba	Taproot	Rhizosphere	10	6–6.5
Carrot	*Daucus carota* subsp. *sativus*	Taproot	Rhizosphere	13	6–6.5
Parsnip	*Pastinaca sativa*	Taproot	Rhizosphere	10	5.5–6
Sunroot	*Helianthus tuberosus*	Tuber	Rhizosphere	11	5.8–6.3
Bell pepper	*Capsicum annuum*	Fruit	Carposphere	10	4.7–5.2
Cucumber	*Cucumis sativus*	Fruit	Carposphere	11	6.4–6.7
Tomato	*Solanum lycopersicum*	Fruit	Carposphere	10	4–5
Cabbage	*Brassica oleracea* var. capitata	Leaves	Phyllosphere	10	5.9–6.0
Leek	*Allium ampeloprasu*m var. porrum	Stem and leaves	Phyllosphere	10	6.2–6.5
Fennel	*Foeniculum vulgare*	Stem and leaves	Phyllosphere	10	5.9–6.2
Green asparagus	*Asparagus officinalis* subsp. *officinalis*	Modified stem	Phyllosphere	9	6.0–6.2

^*a*^ Replicates are biological replicates. Suc: sucrose; Glc: glucose; Fru: fructose; Inu: inulin.

## MATERIALS AND METHODS

### Spontaneous vegetable fermentations and sampling

Vegetables (Table 1) were sliced and fermented in 2.5% (wt/vol) NaCl solution, following a previously established protocol for carrot fermentation (12). For each vegetable, a minimum of nine independent fermentation replicates were prepared. Fermentations were conducted in Weck jars of 250 mL under controlled conditions, within a temperature-controlled room at 20°C. Depending on vegetable size, a single fermentation could contain material from multiple individual plants, while for larger vegetables, multiple fermentation could be prepared from a single plant. Samples (400 µL) were taken on days 7 and 14 from the same fermentation, snap-frozen in liquid nitrogen, and stored at −80°C for subsequent RNA extraction to determine the active community composition.

### Varying salt and CO_2_ levels in lab-controlled vegetable fermentations

To determine the influence of the addition of salt and CO_2_ on the progression of vegetable fermentations, carrot juice fermentation was monitored in biological triplicates over a 28-day period. Carrots were rinsed with tap water and processed in a Solis Juice Fountain Pro Type 843 centrifuge. Varying salt concentrations were added to the fermentation of 0%, 1.25%, or 2.5% (wt/vol) NaCl. Carbon dioxide (CO_2_) was introduced by saturating the samples using a standard home kitchen SodaStream (SodaStream), and the fermentations were divided into 250 mL Weck jars. To minimize interference, individual temporal samples (400 µL) were collected from separate Weck jars at timepoints 0, 2, 3, 7, 14, and 28 days. Each sample was immediately snap-frozen in liquid nitrogen and kept at −80°C for subsequent RNA extraction.

### V4-16S rRNA amplicon sequencing of the active microbial fraction

Total RNA was extracted using the RNeasy PowerMicrobiome kit (Qiagen, Germany), which included an on-column DNase digestion step. After RNA extraction, the absence of DNA was checked using a DNA-dependent polymerase for PCR on the 16S rRNA region (with 27F and 1492R) (38). The lack of PCR product was confirmed on a 1% agarose gel. Complementary DNA (cDNA) was synthesized using Readyscript (Sigma-Aldrich), and 16S rRNA amplicon sequencing was performed as previously described (12).

### Data analysis

The sequencing results were processed with the DADA2 package, version 1.22.0 (39), as previously described (40). In short, reads were classified up to genus level using a combined database from GTDB (version r207) (41) and the SILVA ribosomal RNA gene database (SILVA SSU 138.1) (42), which already included the novel *Lactobacillales* taxonomy (37). Notably, a suffix behind the genus name, for instance Lactococcus_A, indicates that, according to the GTDB, the taxon it was named after (without the suffix) was too diverse and/or not monophyletic. In this case, Lactococcus_A refers to the *Lactococcus carnosus* clade.

Subsequent data analysis and interpretation were executed in the R environment using tidytacos (github.com/LebeerLab/tidytacos, “tidy Taxonomic Compositions”) (43), which builds on tidyverse (44) specifically for the exploration of microbial community data. In addition, it includes multidiffabundance (45) (github.com/thiesgehrmann/multidiffabundance/) (45), which builds on various tools for microbiome analysis, including differential abundance tools ALDEx2 (46), ANCOM-BC2 (47), corncob (48), DESeq2 (49), limma (50), lmclr (45), maaslin2 (51), and ZicoSeq (52). Maaslin2 was used to depict the effect sizes (equivalent to log_2_ fold change), which represent the magnitude and direction of association between taxa and variables. When multiple testing was performed, *P* values were adjusted for false discovery rates. Multi-differential abundance testing interpretable associations across multiple factors rather than relying on a single test, which could falsely represent the true directionality and significance as discussed in Nearing et al (53).

## RESULTS

### *Leuconostoc* dominance is conserved across a wide variety of vegetable fermentations

**Fig 1:**
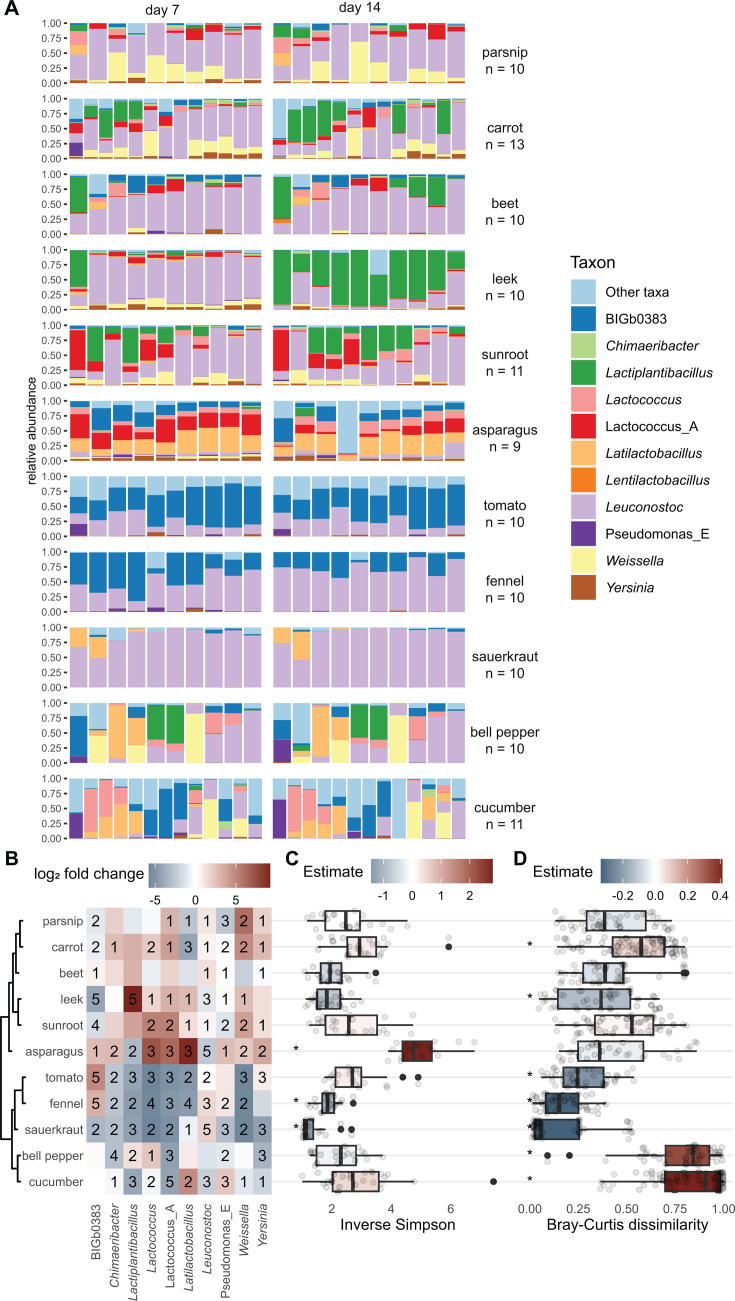
Active bacterial community structure of different vegetables fermented with 2% salt added. Vegetable fermentations were ordered based on a hclust on the Maaslin2 effect sizes, representing the magnitude and direction of association between taxa and vegetables. (**A**) Overview of the microbial community composition of the different vegetable fermentations on day 7 and day 14, *n* = number of biological replicates. The bar plot illustrates genus relative abundances in samples from day 7 and 14. (**B**) Differential abundances of the top 11 most dominant taxa across all fermentations assessed with 8 different differential abundance metrics for each vegetable tested against the collective group. Color shows maaslin2 effect size. Number represents the total number of statistical tests that were significant with FDR corrected *P* value < 0.05. (**C**) Alpha diversity for each vegetable. (**D**) Pairwise Bray-Curtis dissimilarity within vegetables. The color shows the direction of the effect size compared to all other vegetables, *P value < 0.05.

Spontaneous vegetable fermentations offer a simplified yet dynamic system to study ecological assembly processes. In this study, we examined how different vegetable substrates shaped microbial community structure and succession by fermenting 11 commonly consumed vegetables (Table 1). Each vegetable was fermented in 9–13 independent jars (biological replicates), sampled at two time points (day 7 and 14), resulting in 228 samples. All fermentations were set up in a standardized way, with the addition of 2% NaCl, and sampled after 7 and 14 days for microbial community analysis (Fig. 1A). After 14 days, green asparagus showed the highest alpha diversity (inverse Simpson: 4.92 ± 0.76) (Fig. 1C) among all fermented vegetables tested, whereas sauerkraut and fennel showed the lowest alpha diversity (inverse Simpson: 1.33 ± 0.45 and 1.86 ± 0.37, respectively).

LAB were highly prevalent and abundant in most of the fermentations studied, with *Leuconostoc* emerging as the most abundant genus found in 117 of 228 fermentation samples analyzed. Other prominent genera included *Lactiplantibacillus* (29/228), *Latilactobacillus* (18/228), and *Weissella* (10/228). Notably, an uncharacterized *Enterobacteriaceae* taxa BIGB0383 belonging to the family *Enterobacteriaceae* was the most abundant genus in 35 of the 232 fermentation samples and significantly more abundant in tomato and fennel.

Microbial diversity and composition varied substantially between vegetables. The type of vegetable substrate used significantly influenced the overall bacterial composition across all vegetable fermentations. Specifically, the region of the plant (carposphere, phyllosphere, or rhizosphere) explained 6.61% of the variance on day 7 and 9.56% on day 14 (*P* < 0.05, adonis, PERMANOVA, 1,000 iterations). More importantly, the specific vegetable fermented could explain 22.06% on day 7 and 22.68% on day 14. Since the overall community composition was not significantly different between time points across all vegetables, only data from day 14 were included in the subsequent analyses (*R*^2^ = 0.0017, *P* = 0.8).

To more specifically explore the differences in microbial community compositions between the different vegetables, we performed a differential abundance analysis using eight statistical methods (Fig. 1B). While most fermentations were dominated by LAB, the most predominant distinction observed on day 14 was that fermented leek showed a higher abundance of *Lactiplantibacillus* (average relative abundance 66.9%) and lower abundance of BIGB0383 (0.27%) compared to the other vegetables. This analysis confirms that BIGB0383 was more abundant in all tomato (49.87%) and fennel fermentations (25.20%) and was also present in some cucumber (15.07%) and asparagus fermentations (14.9%), albeit at lower abundances (Fig. 1B). Fermented green asparagus had significantly higher abundances of *Yersinia* (3.9%), while sauerkraut and bell pepper had a lower abundance of this potential pathogen (not detected and 0.8%, respectively). Remarkably, fermented green asparagus showed a significantly higher abundance of *Latilactobacillus* (27.0%)*, Lactococcus* (12.9%), and lower *Lactiplantibacillus* (1.53%) compared to all other vegetables (Fig. 1B). Within-vegetable variation was assessed by calculating pairwise Bray-Curtis dissimilarity values (Fig. 1D). These analyses confirmed that cucumbers varied most across biological replicates, while sauerkraut fermentations appeared most similar.

Despite these differential abundance profiles and differences in overall composition, a consistent pattern observed across most fermentations was that LAB, and especially *Leuconostoc*, were dominant.

### Salt reduction slows down LAB dominance and increases *Enterobacterales* abundance

**Fig 2:**
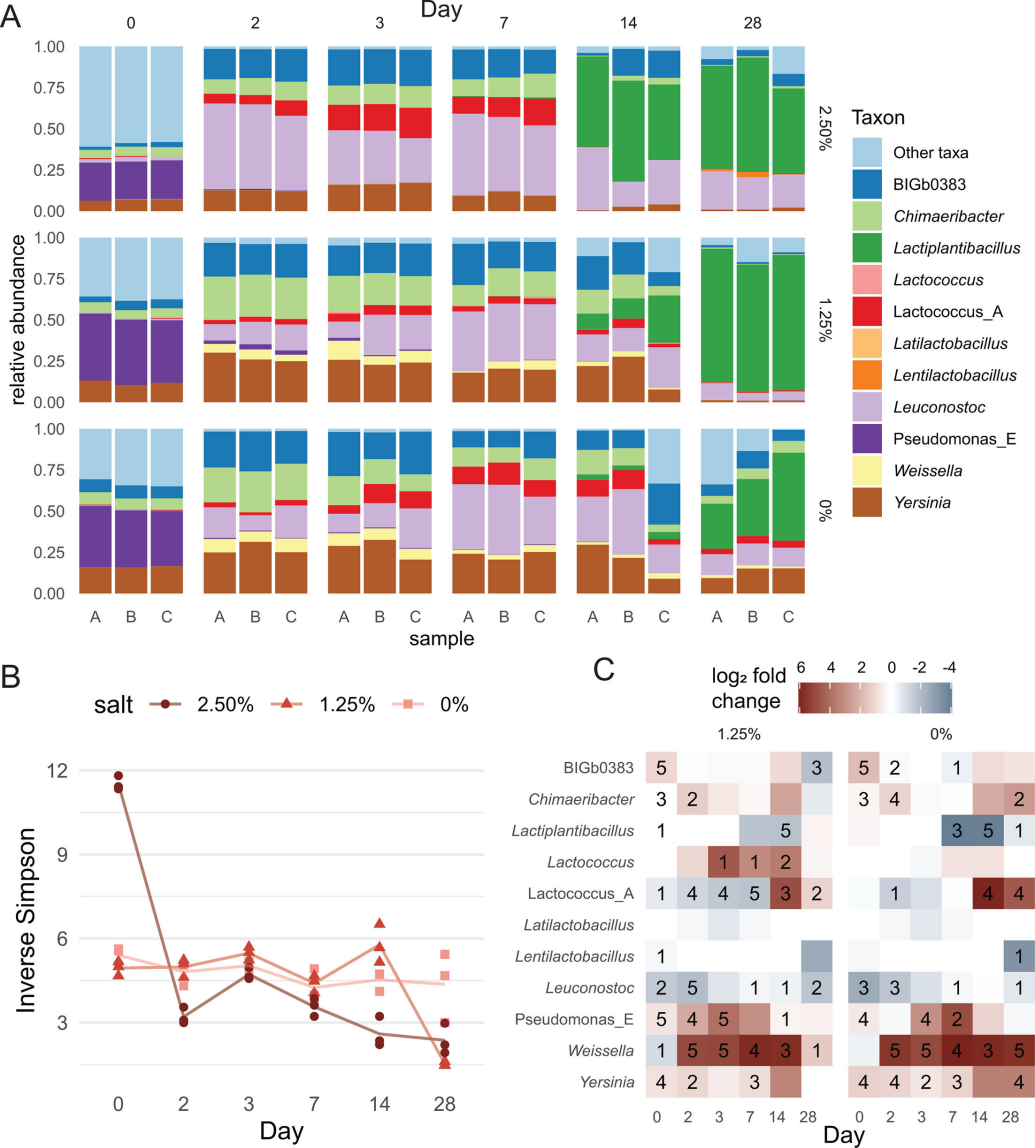
Impact of salt concentration on carrot juice fermentation. (**A**) Bar plot illustrating carrot juice fermentations with three biological replicates of three different salt conditions over time. (**B**) Alpha diversity calculated with inverse Simpson over time compared to the samples with a reference salt concentration of 2.50% NaCl. Different shades of red represent the different salt (NaCl) concentrations used. (**C**) Multi-differential abundance of lower-salt fermentations compared to 2.5% NaCl: the number refers to the number of pipelines (ALDEx2, ANCOM-BC2, corncob, DESeq2, limma, lmclr, maaslin2, and ZicoSeq) that indicate a significant difference. Log2 fold change is shown determined with Maaslin2 to indicate direction and magnitude (*P* values were corrected for false discovery rate FDR, *P < 0.05).

While the vegetable substrate plays a key role in shaping microbial communities, environmental factors must also be considered in these man-made ecosystems. Among these, salt addition is one of the primary interventions used to steer microbial dynamics in fermented vegetables. To better understand its influence, we investigated how different salt concentrations influence active microbial community composition and the potential suppression of spoilage organisms and pathogens. We selected carrot juice fermentation as a model system based on our prior experience with this ecosystem and its practical advantages in the laboratory. Its liquid nature makes it easier to handle and sample under controlled conditions (12, 20). Carrot juice fermentations were set up with three salt concentrations, 2.5%, 1.25%, and 0% NaCl, to mimic standard conditions, lower salt concentration, and no salt conditions, respectively. Samples were collected over a 28-day period. As observed in the various vegetable fermentations, LAB were the most predominant members with low variation between different biological replicates. The microbial community initially consisted primarily of heterofermentative *Leuconostoc* and gradually shifted to a more homofermentative *Lactiplantibacillus-*dominated community, depending on the salt concentration (Fig. 2A).

Alpha diversity was used as a proxy for fermentation consistency, with lower diversity indicating a more LAB-dominated community (Fig. 2B). Compared to 2.5% NaCl, significantly higher alpha diversity was observed up to day 14 in fermentations with 0% and 1.25% salt. By day 28, the 1.25% and 2.5% salt groups had the same alpha diversity, suggesting that 1.25% NaCl fermentations progressed more slowly but eventually reached a similar *Lactiplantibacillus*-dominated state. In contrast, diversity remained higher in the no-salt group, representing lower LAB dominance.

Differential abundance analysis revealed that on day 0, fermentations with all salt concentrations displayed high levels of *Pseudomonas*, *Yersinia*, *Chimaeribacter*, and the unclassified *Enterobacterales* taxon BIGb0383, though at varying abundances. When 2.5% salt was added, *Leuconostoc* became dominant (>50% relative abundance) after 2 days, while under 1.25% and 0% salt conditions, this shift occurred after 7 days (Fig. 2C). A similar delay was observed for *Lactiplantibacillus*, which dominated by day 14 in 2.5% salt, but only by day 28 in lower salt conditions. Notably, in the 0% salt condition, only 2 out of 3 fermentations reached *Lactiplantibacillus* dominance by day 28. 1.25% and 0% salt conditions also showed prolonged presence of potential spoilage taxa such as *Yersinia*, *Pseudomonas_E*, *Chimaeribacter*, and *BIGb0383. Weissella* was more abundant at nearly all timepoints in 1.25% salt conditions, while *Lactococcus_A* increased later in fermentation. Taken together, these results indicate that reducing salt hampers LAB succession and allows spoilage-associated taxa to persist longer.

### CO_2_ addition under low salt conditions restores rapid LAB community domination

**Fig 3:**
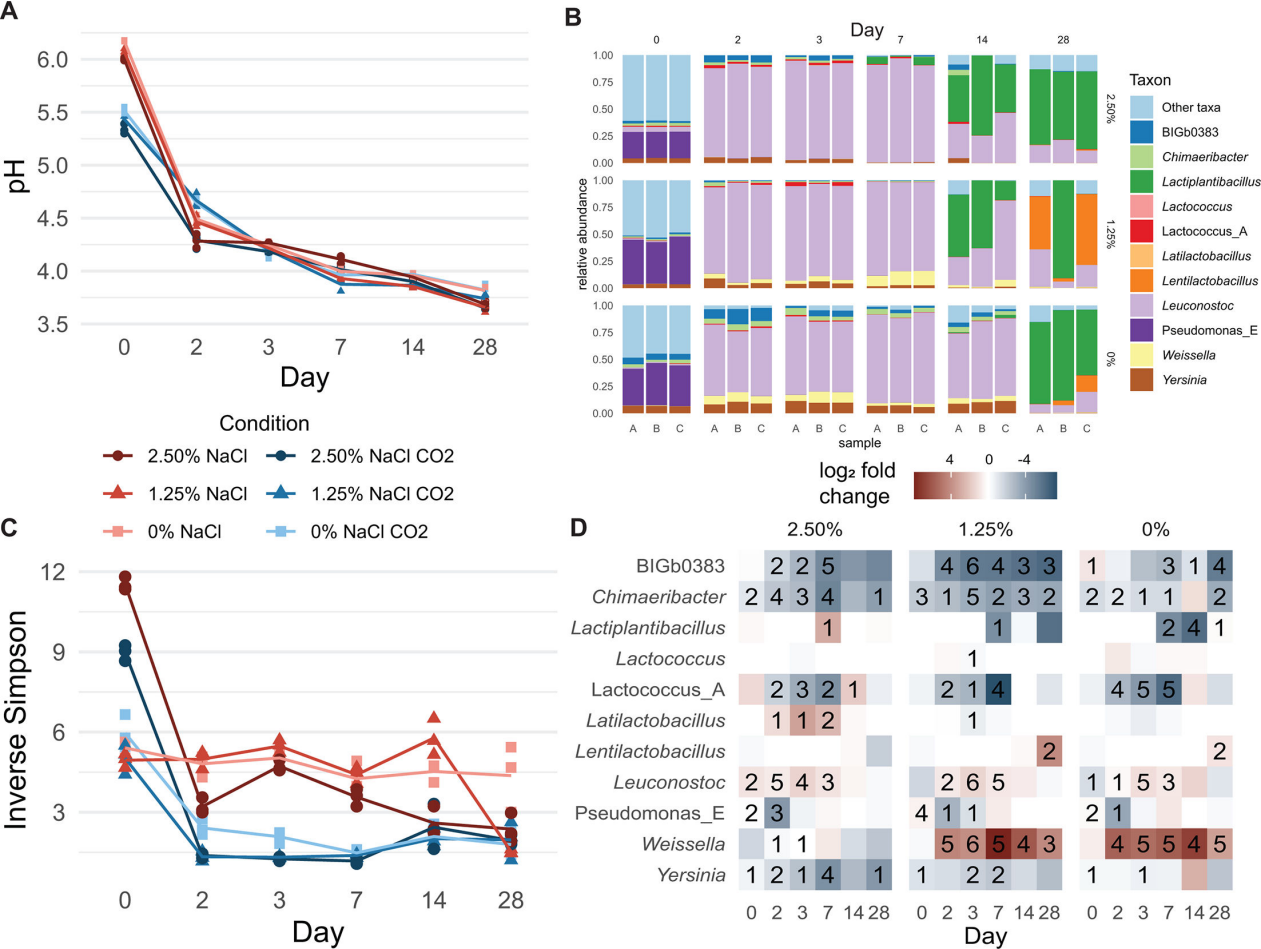
Influence of CO_2_ on microbial community dynamics in carrot juice fermentations. Each fermentation has three biological replicates. Shades of red represent the fermentations with CO_2_ treatment (CO_2_), while shades of blue represent the fermentations without CO_2_ injected. (**A**) pH of carrot juice fermentations with various salt concentrations and the addition of CO_2_ before the start of fermentation. (**B**) Bar plot illustrating the microbial community after the addition of CO_2_ in the fermentation under different salt concentrations, for the control, see Fig. 2A. (**C**) Alpha diversity over time compared to the reference salt concentration of 2.50% NaCl. (**D**) Multi-differential abundance of CO_2_-injected fermentations compared to 2.5% NaCl without CO_2_: the number refers to the number of pipelines (ALDEx2, ANCOM-BC2, corncob, DESeq2, limma, lmclr, maaslin2, and ZicoSeq) that indicate a significant difference. Log2 fold change is shown determined with Maaslin2 to indicate direction and magnitude (“*” = FDR corrected *P* < 0.05).

When lower salt concentrations are desired, alternative strategies are required to ensure LAB dominance. Therefore, we next investigated the potential of CO₂ as a selective intervention to support LAB-driven fermentation. Hereto, carrot juice fermentations were performed with or without prior CO_2_ injection. Notably, CO_2_-treated fermentations started at a lower pH (6.08 ± 0.03 vs 5.43 ± 0.03; *P* < 0.05; Fig. 3A), suggesting that CO_2_ injection led to an initially more acidic environment. Despite this initial decrease, the overall pH trajectory during the fermentation was not altered in later stages. Interestingly, by day 2, pH values in CO₂-treated fermentations were slightly higher than in non-treated controls (*P* < 0.05), suggesting a small temporary buffering effect despite the initially lower pH. CO₂ injection significantly influenced early fermentation dynamics. Similar to the fermentations without CO_2_-injection, the communities were dominated by *Leuconostoc*, followed by the homofermentative *Lactiplantibacillus*. However, in the later stages, some samples with lower salt concentration contained *Lentilactobacillus* (Fig. 3B). Alpha diversity remained significantly lower in CO₂-treated fermentations compared to controls until day 7, after which no differences were observed (Fig. 3C).

Differential abundance analysis at the genus level showed that CO₂ injection led to a significant reduction in *Yersinia, Chimaeribacter*, and BIGb0383 on days 3 and 7 even in the absence of salt (Fig. 3C and D). In parallel, *Leuconostoc* was significantly more abundant in CO₂-treated fermentations at these timepoints. Similar to the non-treated fermentation, *Weissella* was also increased in low-salt conditions that were treated with CO_2_. Overall, CO₂ consistently promoted a LAB-dominated, low-*Enterobacterales* community structure.

These findings demonstrate that CO₂ injection at the start of vegetable fermentations can effectively steer microbial succession, suppress spoilage-associated taxa, and enhance LAB dominance, especially under low- or no-salt conditions. This highlights CO₂ as a promising tool to modulate fermentation ecosystems toward safer and more desirable outcomes.

## DISCUSSION

This study demonstrates how fermented vegetables are dominated by LAB in a robust and reproducible manner. By systematically varying environmental parameters, including substrate type, salt concentration, and CO₂ levels, we observed reproducible patterns in LAB dominance and demonstrated how ecological factors can be leveraged to steer microbial communities. These findings reinforce the value of fermented food systems as experimentally accessible platforms for testing ecological hypotheses in complex yet controllable environments.

Understanding bacterial community dynamics and competitive interactions is essential for optimizing the flavor, safety, and quality of fermented vegetables. While previous studies have typically focused on individual vegetables (12, 17, 21, 54, 55), we performed a standardized longitudinal analysis examining the active community through RNA-based amplicon sequencing across 11 different vegetables. However, it should be noted that rRNA-based sequencing provides a proxy for metabolic activity and presence but does not mean true viability, as rRNA can persist after cell death (12, 56). Specifically, standardized handling was shown to be important as previous work documented that fermentation dynamics were influenced by handling and cutting types of fresh vegetables (57).

In this study, the specific vegetable was the strongest predictor of overall bacterial composition. The region of the plant (carposphere, phyllosphere, or rhizosphere) also contributed, but to a lesser extent. Although biological replicate fermentation of the same vegetable was generally grouped together, variation was observed between samples of the same vegetable, as reflected by the higher Bray-Curtis dissimilarities (Fig. 1D). Such within-vegetable variation may result from stochastic effects and ecological drift, particularly because initial LAB abundances in raw vegetables are typically low (58). Under these conditions, random differences in substrate microbial population, early colonization, and selection pressure can lead to divergent communities (59). Most fermentations were consistently dominated by LAB, predominantly *Leuconostoc*. Remarkably, tomato and fennel fermentations were dominated by the uncharacterized Enterobacteriaceae taxon BIGB0383, which has also been detected in the *Caenorhabditis elegans* gut (60). Given its close phylogenetic relationship to *Pseudescherichia*, an environmental microorganism, its functionality during fermentation remains ambiguous (61, 62). However, it is important to note that, due to the limitations of the current approach, taxonomic identification was only possible at the genus level. Species-level or strain-level resolutions would be needed to fully characterize this unexpected member of the fermented fennel and tomato microbial community. Additionally, *Enterobacterales* are common early colonizers in spontaneous plant fermentations, and their role remains nuanced (13). In future research, more focus could be given to correlating the microbial profiling with chemical and metabolic measurements of the raw and fermented vegetables, enabling a clearer understanding of how substrate-derived metabolites and pH dynamics drive ecological transitions during various vegetable fermentations.

We then explored the importance of externally controllable factors, such as salinity, on steering vegetable fermentations. Reducing salt concentration clearly shifted the temporal microbial community dynamics. Lower salt concentrations were associated with higher alpha diversity compared to the reference condition of 2.5% NaCl, accompanied by the presence of less favorable *Enterobacterales*, including the genera *Yersinia* and *Chimaeribacter,* as well as the non-*Enterobacterales* genus *Pseudomonas*. A slower microbial succession from heterofermentative *Leuconostoc* to homofermentative *Lactiplantibacillus*-dominated communities was also observed in lower salt concentrations. This trend partially aligned with findings in kimchi, which reported a lower abundance of homofermentative *Latilactobacillus* at lower salt concentrations although their study was limited to a single replicate (21). Others have also observed elevated *Enterobacteriaceae* counts at lower salt concentrations in fermented cabbage (34), but they only measured colony-forming units of specific taxa of interest while our sequencing approach of the active fraction (RNA) allowed for a more comprehensive overview. Together, our findings clearly demonstrate that reducing salt concentrations not only increases the abundance of unwanted *Enterobacteriaceae* but also prolongs their presence, potentially leading to the accumulation of undesirable compounds such as biogenic amines (63).

We also investigated atmospheric conditions by focusing on CO₂ addition as an alternative strategy to modulate this ecosystem. Saturation of the fermentation matrix with CO₂ in carrot juice successfully induced a more rapid decline of *Enterobacterales* and a clear dominance of *Leuconostoc*. While an immediate pH reduction was observed following CO₂ introduction, acidification alone could not fully account for the suppression of *Enterobacterales*, as these taxa persisted at much lower pH in control fermentations with 2.5% NaCl. Nevertheless, even a slight pH reduction can influence relative growth rates without causing complete inhibition, reducing the initial dominance of *Enterobacterales* (64). Prior studies have reported the antimicrobial effects of both high-pressure and dissolved CO₂ (36, 65–68), supporting the hypothesis that CO₂ directly contributed to the observed shifts in community structure. Our results demonstrated that CO₂ addition consistently decreased alpha diversity, suppressed the growth of potential spoilage organisms, and enhanced the predominance of beneficial *Lactobacillales* across different salt concentrations. Shifts within the LAB community were also observed, including an increase in *Weissella* at lower salt levels. In the final stage, *Lentilactobacillus* also appears; however, its role remains context-dependent, as there are limited case studies of spoilage in cucumbers (69, 70). However, a low but non-negligible abundance of *Yersinia* was present in various conditions, including the no-salt CO_2_-treated fermentation and the 2.5% salt without CO_2_ injection fermentation. This suggests that a combinatorial approach could be beneficial for engineering this ecosystem to minimize the presence of undesirable taxa as early and effectively as possible. Additionally, the addition/flushing of CO_2_ should be validated in various vegetable fermentations to reduce possible side effects like the bloater effect in cucumbers (71). Overall, CO₂ addition emerges as a promising strategy to enhance fermentation safety while reducing salt content, opening opportunities for robust and healthier fermented vegetable products.

The results from our current study can be used to explore questions about the ecological mechanisms driving LAB succession in vegetable fermentations. While high salinity has long been considered a key selective pressure favoring LAB, our results suggest that other environmental factors, such as the addition of CO_2_, can be used to modulate the microbial community assembly. Of note, heterofermentative LAB, such as *Leuconostoc*, also produce CO₂ as a metabolic byproduct, which may positively influence community dynamics during fermentation.

In summary, this study addressed three key knowledge gaps in the field of vegetable fermentation. First, it showed a systematic LAB dominance of the microbial community across a wide range of vegetable types. Second, it demonstrated the essential role of salt in microbial community dominance and succession. Third, it revealed the potential of CO₂ addition to simultaneously restore natural microbial dynamics and enable food-safe salt reduction in vegetable fermentations. Given its promising impact, further research into the scalability and robustness of CO₂ injection in larger fermentation systems is warranted. More broadly than merely food fermentation applications, this study illustrates that targeted manipulation of environmental parameters can be used to steer microbial ecosystems.

## Supplementary Material

Reviewer comments

## Data Availability

Amplicon sequencing data are available on ENA (PRJEB98055).

## SUPPLEMENTAL MATERIAL

OPEN PEER REVIEW reviewer-comments.pdf An accounting of the reviewer comments and feedback.

ASM does not own the copyrights to Supplemental Material that may be linked to, or accessed through, an article. The authors have granted ASM a non-exclusive, world-wide license to publish the Supplemental Material files. Please contact the corresponding author directly for reuse.
