# Supplementary material for: From diversity to dominance: how salt and CO₂ shape LAB-dominated ecosystems in vegetable fermentations
Source: Microbiol Spectr. 2026 Jun 30;14(8):e03578-25. doi: 10.1128/spectrum.03578-25 (PMC13435955; doi:10.1128/spectrum.03578-25)
Supplement: Supplementary file 1 [file reviewer-comments.pdf]

AMERICAN SOCIETY FOR MICROBIOLOGY | Microbiology Spectrum

From Diversity to Dominance: How Salt and CO_2 Shape LAB-dominated Ecosystems in Vegetable Fermentations

Tom Eilers, Tim Van Rillaer, Stijn Wittouck, Ines Tuyaerts, Katrien Michiels, Maline Victor, Thies Gehrmann, Peter Bron, Wannes Van Beeck, and Sarah Lebeer

Corresponding Author(s): Sarah Lebeer, Universiteit Antwerpen

Review Timeline:

Submission Date:	February 19, 2026
Editorial Decision:	April 10, 2026
Revision Received:	May 29, 2026
Accepted:	June 7, 2026

Editor: Erik Hom

Reviewer(s): Disclosure of reviewer identity is with reference to reviewer comments included in decision letter(s). The following individuals involved in review of your submission have agreed to reveal their identity: Luca Cocolin (Reviewer #2)

Transaction Report:

(Note: With the exception of the correction of typographical or spelling errors that could be a source of ambiguity, letters and reports are not edited. The original formatting of letters and referee reports may not be reflected in this compilation.)

DOI: https://doi.org/10.1128/spectrum.03578-25

Re: Spectrum03578-25 (From Diversity to Dominance: How Salt and CO_2 Shape LAB-dominated Ecosystems in Vegetable Fermentations)

Dear Prof. Sarah Lebeer:

Thank you for the privilege of reviewing your work. Below you will find my comments, instructions from the Spectrum editorial office, and the reviewer comments.

Please carefully address the reviewers' comments and make sure you deposit your data at NCBI and provide accession numbers in your manuscript--and include a Data Availability statement. Please summarize your changes to me in your cover letter upon submitting your revision.

Please return the manuscript within 60 days; if you cannot complete the modification within this time period, please contact me. If you do not wish to modify the manuscript and prefer to submit it to another journal, notify me immediately so that the manuscript may be formally withdrawn from consideration by Spectrum.

Revision Guidelines
To submit your modified manuscript, log into the submission site at https://spectrum.msubmit.net/cgi-bin/main.plex. Go to Author Tasks and click the appropriate manuscript title to begin. The information you entered when you first submitted the paper will be displayed; update this as necessary. Note the following requirements:

• Upload point-by-point responses to the issues raised by the reviewers in a file named "Response to Reviewers," NOT in your cover letter.
• Upload a compare copy of the manuscript (without figures) as a "Marked-Up Manuscript" file.
• Upload a clean .DOC/.DOCX version of the revised manuscript and remove the previous version.
• Each figure must be uploaded as a separate, editable, high-resolution file (TIFF or EPS preferred), and any multipanel figures must be assembled into one file.
• Any supplemental material intended for posting by ASM should be uploaded with their legends separate from the main manuscript. You can combine all supplemental material into one file (preferred) or split it into a maximum of 10 files with all associated legends included.

For complete guidelines on revision requirements, see our Submission and Review Process webpage. Submission of a paper that does not conform to guidelines may delay acceptance of your manuscript.

Data availability: ASM policy requires that data be available to the public upon online posting of the article, so please verify all links to sequence records, if present, and make sure that each number retrieves the full record of the data. If a new accession number is not linked or a link is broken, provide Spectrum production staff with the correct URL for the record. If the accession numbers for new data are not publicly accessible before the expected online posting of the article, publication may be delayed; please contact production staff (Spectrum@asmusa.org) immediately with the expected release date.

Publication Fees: For information on publication fees and which article types are subject to charges, visit our website. If your manuscript is accepted for publication and any fees apply, you will be contacted separately about payment during the production process; please follow the instructions in that e-mail. Arrangements for payment must be made before your article is published.

ASM Membership: Corresponding authors may join or renew ASM membership to obtain discounts on publication fees. Need to upgrade your membership level? Please contact Customer Service at Service@asmusa.org.

The ASM Journals program strives for constant improvement in our submission and publication process. Please tell us how we can improve your experience by taking this quick Author Survey.

Thank you for submitting your paper to Spectrum.

Sincerely,
Erik Hom
Editor
Microbiology Spectrum

Reviewer #1 (Comments for the Author):

This reviewer was quite happy with the initial submission and comments were adequately addressed. I remain with only few minor comments:

- It is somewhat unclear what the experimental unit is when experimenting with plants as source of fermentation microbes: This can be defined as the individual plant, or as a batch of plants that were grown on the same field (or purchased in the same (super)market at the same time). There is no right or wrong answer but: The response to reviews indicates that "plant material was sourced from different farms or stores" but the manuscript does not. If Table 1 can be modified to include the # of farms or stores in brackets after the number of replicates, this would clarify replication better.

line 385. This reviewer prefers simple answers and a reduced pH is a perfectly fine explanation to the faster dominance of Leuconostoc. To illustrate: a pH of 5.5 is nowhere close to inhibit even the most acid sensitive Enterobacteriaceae. To grow from initial numbers (10^4) to cell counts relevant in fermentations (10^7) about 10 generations. Leuconostoc requires additional generations as they occur in lower initial cell counts. Even a modest reduction of the generation time of Enterobacteriaceae (e.g. 30 to 45 min), which is likely achieved by acidification from pH 6.0 to 5.5, with an unaltered generation time of Leuconostoc could lead to initial dominance of leuconostoc instead of Enterobacteriaceae. The observation that a modest reduction of the pH shortcuts initial dominance of Enterobacteriaceae is known for olives (where vinegar is added to reduce the pH to 5 - 5.5) and for sponge dough fermentations (where baker's yeast consumes oxygen and reduces the pH to ~ 5.5 by CO2 production). This is not well documented in the scientific literature, though.
line 348. delete the lower case "f"

Reviewer #2 (Public repository details (Required)):

I'm not sure the datasets were publicly deposited.

From Diversity to Dominance: How Salt and CO_2 Shape LAB-dominated Ecosystems in Vegetable Fermentations

Tom Eilers[1], Tim Van Rillaer[1], Stijn Wittouck[1], Ines Tuyaerts[1], Katrien Michiels[1], Maline Victor[1], Thies Gehrmann[1], Peter A Bron[1], Wannes Van Beeck[*1], Sarah Lebeer*[1]

Affiliations

[1]Department of Bioscience Engineering, Lab of Applied Microbiology and Biotechnology, University of Antwerp, Groenenborgerlaan 171, 2020 Antwerp, Belgium

*These authors jointly supervised this work

Email corresponding author: Sarah.Lebeer@uantwerpen.be

Abstract

Research on microbial ecosystems is often challenging due to high diversity of microbial taxa present and the complexity of controlling environmental variables. To address these challenges, fermented foods are simpler and more reproducible model ecosystems, where both community composition and environmental factors can be more precisely controlled and manipulated. In this study, we focused on fermented vegetables which are typically dominated by lactic acid bacteria (LAB). It is not completely understood why ~~lactic acid bacteria (~~LAB~~)~~ consistently drive the spontaneous fermentation of vegetables such as cabbage and carrots and how variables such as vegetable substrates, salt addition, and carbon dioxide levels can impact microbial community dynamics. Here, we explored the temporal microbial dynamics in standardized fermentations of 11 different vegetables (including beetroot, bell pepper, cabbage, carrot, cucumber, fennel, green asparagus, leek, parsnip, sunroot, and tomato), revealing a consistent dominance of *Leuconostoc* and other LAB. Additionally, we investigated the impact of varying salt concentrations, demonstrating that lower salt levels resulted in a delayed appearance of the typically dominant LAB community, while simultaneously revealing a higher abundance of *Weissella* and various *Enterobacterales* taxa. These effects imposed by reduced salt concentrations were mitigated by CO_2 injection, which reverted the enhanced *Enterobacterales* levels and increased the overall abundance of *Lactobacillales*. This study demonstrates how targeted manipulation of environmental parameters, such as salinity and gas composition, can be used to uncover ecological principles governing microbial succession and community assembly in reproducible fermentation-based model ecosystems.

Importance:

Understanding the ecological principles that shape microbial community assembly is essential for advancing our knowledge of microbial ecosystems. Fermented vegetables, increasingly popular among the general population, provide tractable and reproducible model systems to study microbial succession under controlled environmental conditions. By systematically manipulating variables such as vegetable type, salinity and gas composition, we uncovered the effect of these factors on the microbial dynamics throughout the fermentation. These insights not only contribute to a better understanding of the microbial ecology of these man-made food systems but also suggest directions for novel strategies to optimize fermentation processes for producing faster, safer, and more flavorful foods.

Introduction

Research on microbial ecosystems is often challenging due to the large diversity of taxa and the numerous and often unknown external factors that shape ecosystem development. To better understand the key drivers of microbial dynamics and interactions, simpler, manipulable and reproducible model systems are needed. Fermented foods offer such systems, natural yet tractable ecosystems, in which external parameters can be systematically varied[1]. Their adaptability allows researchers to modulate conditions such as substrate type, salinity, CO_2 levels, temperature, and fermentation duration. This type of mechanistic ecological research not only advances our understanding of microbial community assembly but also informs practical strategies for microbiome engineering in applied contexts.

In recent years, fermented food ecosystems have also gained renewed popularity in society, driven by the growing production and consumption of artisanal and industrial fermented products. This resurgence in Western culture is fueled by their desirable organoleptic properties, their potential to promote sustainability by reducing food waste, and increasing evidence of their health benefits [2–6]. Fermented foods encompass distinct food products, but here we adhere to the scientific definition that fermented foods are ‘foods made through desired microbial growth and enzymatic conversions of food components’ [7]. It is estimated that over 5000 different fermented foods exist globally, which can be divided based on the substrate fermented [8]. In addition to cow’s milk, vegetables form one of the most important groups of substrates of fermented foods [9].

Vegetable fermentation typically begins with chopping or crushing the vegetables, followed by the addition of salt and the exclusion of air by sealing the mixture in airtight jars. These conditions selectively stimulate the growth of a desired subset of the native microbial community, primarily lactic acid bacteria (LAB) [10]. LAB are known for their ability to ferment plant-derived sugars into lactic acid, which lowers pH and inhibits the growth of undesirable microbes, particularly members of the order Enterobacterales[11]. These spoilage-associated taxa typically co-occur with LAB at the onset of fermentation and are linked to the production of off-flavors and potentially harmful compounds such as biogenic amines and toxins[12].

A few selected vegetable fermentations have been extensively studied so far and include sauerkraut [13,14], kimchi [15,16], cucumber [17], and fermented carrot juice [12,18]. Among these fermentations, cabbage and carrot fermentations consistently exhibit a characteristic succession: an initial dominance of heterofermentative LAB, such as *Leuconostoc*, followed by homofermentative genera like *Lactiplantibacillus* and *Latilactobacillus*[12,13,19]. However, it remains unclear whether similar microbial dynamics occur in the fermentation of other vegetables. Moreover, the ecological mechanisms driving

LAB dominance and succession are still poorly understood. Potential drivers include the starting substrate's initial microbial community [20] and nutrient composition, [21], as well as broader environmental influences, akin the concept of 'microbial terroir' in wine fermentation, where geographic and environmental factors shape microbial communities [22]. In this context, it is still unknown whether the location on the plant, i.e. soil-exposed, nutrient-dense rhizosphere and air exposed phyllosphere and carposphere affect the microbial dynamics during fermentation.

Other environmental variables affect and can thus be modulated to influence the microbial community dynamics that occur during the fermentation. Traditionally, variable concentrations of salt, typically ranging from 2-8%, are added to vegetable fermentations [23–25], decreasing the water activity [26], increasing osmotic stress. However, such high salt concentrations are negatively perceived by consumers for organoleptic properties [27], and linked to (presumed) negative health effects of the fermented end product [26,28,29]. Recent studies have explored reducing salt concentrations in traditional fermentations, down to 1.4% in kimchi [19], 0.5% in sauerkraut [30], and 0% in cucumbers [31], while monitoring metabolites and pH dynamics. Although these studies only partially examined the microbial communities, they reported distinct shifts in microbial composition and fermentation profiles. However, comprehensive investigations into the complete removal of salt and its effects on the full microbial community remain scarce.

Atmospheric conditions, including the presence of carbon dioxide (CO_2), represent another environmental factor influencing microbial community dynamics during fermentation. Carbon dioxide (CO_2) has been shown to inhibit a broad range of spoilage organisms. For instance, in liquid broth experiments where CO_2 was introduced via gas packaging, concentrations of 2000 mg/L CO_2 completely inhibited several Gram-negative bacteria such as *Pseudomonas fluorescens*, *Photobacterium phosphoreum*, *Shewanella putrefaciens*, and *Aeromonas hydrophila*. Gram-positive such as *Brochothrix thermosphacta* and *Bacillus circulans* [32] were also inhibited, albeit to a lesser extent at the same concentration. In contrast, the LAB *L. sakei* exhibited only a moderate reduction in growth rate at concentrations up to 2500 mg/L CO_2, suggesting that CO_2 may act as a selective pressure favoring LAB dominance. Interestingly, CO_2 is also naturally produced during vegetable fermentation by heterofermentative LAB such as *Leuconostoc* spp., yet its potential role in shaping microbial succession remains unexplored. Understanding how atmospheric composition influences microbial ecology could open new avenues for steering fermentation dynamics, particularly in low- or no-salt conditions.

To clarify how substrate characteristics, salinity, and atmospheric conditions (in particular CO_2) shape microbial community assembly and succession in plant-associated fermentation ecosystems, we

conducted a systematic investigation under standardized conditions. Eleven vegetables (Table 1) coming from distinct plant regions, carposphere (e.g. tomato), phyllosphere (e.g. cabbage), and rhizosphere(e.g. carrots and sunroot), were fermented, resulting in 228 samples collected at two timepoints. The active microbial communities were analyzed at the genus level using the updated *Lactobacillaceae* taxonomy [33]. The specific effects of salt and CO_2 were examined using carrot juice fermentations as a controlled model system, enabling detailed analysis of their impact on microbial succession and LAB dominance.

Materials and Methods

Spontaneous vegetable fermentations and sampling

Vegetables (Table 1) were sliced and fermented in 2.5% w/v NaCl solution, following a previously established protocol for carrot fermentation[12]. Fermentations were conducted under controlled conditions, within a temperature-controlled room at 20°C. 400 µl of sample were taken on day 7 and 14, and frozen in liquid nitrogen and stored at -80°C for subsequent RNA extraction to measure the active community.

Table 1 Overview of the various vegetables used in this project.

Vegetable	Latin name	Plant organ	Region of plant
Beetroot	*Beta vulgaris* var. Ruba	Taproot	Rhizosphere
Carrot	*Daucus carota* subsp. *sativus*	Taproot	Rhizosphere
Parsnip	*Pastinaca sativa*	Taproot	Rhizosphere
Sunroot	*Helianthus tuberosus*	Tuber	Rhizosphere
Bell pepper	*Capsicum annuum*	Fruit	Carposphere
Cucumber	*Cucumis sativus*	Fruit	Carposphere
Tomato	*Solanum lycopersicum*	Fruit	Carposphere
Cabbage	*Brassica oleracea* var. capitata	Leaves	Phyllosphere
Leek	*Allium ampeloprasum* var.porrum	Stem and leaves	Phyllosphere
Fennel	*Foeniculum vulgare*	Stem and leaves	Phyllosphere
Green asparagus	*Asparagus officinalis* subsp. *officinalis*	Modified stem	Phyllosphere

Varying salt and CO_2-levels in lab-controlled vegetable fermentations

To determine the influence of addition of salt and CO_2 on the progression of vegetable fermentations, carrot juice fermentation was monitored over a 30-day period. Carrots were rinsed with H_2O and were processed with Solis Juice Fountain Pro Type 843 centrifuge. Varying salt concentrations were added to the fermentation of 0%, 1.25% or 2.5% w/v NaCl. Carbon dioxide (CO_2) was introduced by saturating the samples using a standard home kitchen SodaStream (SodaStream), and the fermentations were divided into 0.25 l Weck jars. To minimize interference, individual temporal samples were collected from separate Weck jars, at timepoints 0, 2, 3, 7, 14, and 28 days. Each 400 µl sample was immediately frozen in liquid nitrogen and kept at -80°C for subsequent RNA extraction.

V4-16S rRNA amplicon sequencing of the active microbial fraction

Total RNA was extracted using the RNeasy PowerMicrobiome kit (Qiagen), which included an on-column DNase digestion step. After RNA extraction, the absence of DNA was checked using a PCR on the 16S region (with 27F and 1492R), and the lack of PCR product was confirmed on a 1% agarose gel. Complementary DNA (cDNA) was synthesized using Readyscript (Sigma-Aldrich), and 16S rRNA amplicon sequencing was performed as previously described in De Boeck et al. 2020 [12].

Data analysis

The sequencing results were processed with the DADA2 package, version 1.22.0 [34], as described in Lebeer et al (2023). However, reads were classified up to genus level using a combined database from GTDB (version r207 [35]) and the SILVA ribosomal RNA gene database (SILVA SSU 138.1 [36]), which already included the novel *Lactobacillales* taxonomy as described by Zheng et al (2020) [33]. Notably, a suffix behind the genus name, for instance Lactococcus_A, indicates that according to the GTDB the taxon it was named after (without the suffix) was too diverse and/or not monophyletic. In this case, Lactococcus_A refers to the *Lactococcus carnosus* clade.

Subsequent data analysis and interpretation were executed in the R environment using in-house developed packages including tidytacos (github.com/LebeerLab/tidytacos, "tidy Taxonomic Compositions") [37], which builds on tidyverse [38] specifically for the exploration of microbial community data. In addition, it includes multidiffabundance [39] (github.com/thiesgehrmann/multidiffabundance/) [39], which builds on various tools for microbiome analysis, including differential abundance tools ALDEx2[40], ANCOM-BC2[41], corncob [42], DESeq2[43], limma[44], lmclr [39], maaslin2[45], and ZicoSeq [46]. When multiple testing was performed, p-values were adjusted for false discovery rates.

Results

Leuconostoc-dominance is conserved across a wide variety of vegetable fermentations

Microbial fermentations offer a simplified yet dynamic system to study ecological assembly processes. In this study, we examined how different vegetable substrates shaped microbial community structure and succession by fermenting 11 commonly consumed vegetables (Table 1). 114 vegetable fermentations were set up in a standardized way, with addition of 2% NaCl and sampled after 7 and 14 days for microbial community analysis (Figure 1A). At the end of these 14day-fermentations, asparagus showed the highest alpha diversity (inverse Simpson: 4.92 ± 0.76, Figure 1C) among all fermented vegetables tested, whereas sauerkraut and fennel showed the lowest alpha diversity (inverse Simpson: 1.33 ± 0.45 and 1.86 ± 0.37, respectively).

LAB appeared highly prevalent and abundant in most of the fermentations studied, with *Leuconostoc* the most abundant genus found in 117 of 228 fermentation samples analyzed. Other prominent gena included *Lactiplantibacillus* (29/228), *Latilactobacillus* (18/228), and *Weissella* (10/228). Notably, an uncharacterized *Enterobacteriaceae* taxa BIGB0383 belonging to the family *Enterobacteriaceae* (Supplementary S1), was the most abundant genus in 35 of the 232 fermentation samples and significantly more abundant in tomato and fennel. Differences in microbial diversity and composition were also observed between the different fermented vegetable substrates studied. An adonis test demonstrated that the type of vegetable substrate used significantly influenced overall bacterial composition across all vegetable fermentations. Specifically, the region of plant (Carposphere, phyllosphere or rhizosphere) explained 6.61% of the variance on day 7 and 9.56% on day 14. More importantly, the specific vegetable fermented could explain 22.06% on day 7 and 22.68% on day 14. Since the overall community composition was not significantly different between time points across all vegetables, only data from day 14 were included in the remainder of the analyses (R^2 = 0.0017, p=0.8).

To more specifically explore the differences in microbial community between the different vegetables, we performed a differential abundance analysis using eight statistical methods (Figure 1B). While most fermentations were dominated by LAB, the most predominant distinction observed on day 14 was that fermented leek showed a higher abundance of *Lactiplantibacillus* and lower abundance of BIGB0383 compared to the other vegetables. In contrast, BIGB0383 was more abundant in all tomato and fennel fermentations and was also present in some cucumber and asparagus fermentations, albeit at lower abundances (Figure 1B). Fermented green asparagus and tomato had significantly higher abundances of *Yersinia*, while sauerkraut and bell pepper had a lower abundance of this potential pathogen. Remarkably, fermented green asparagus showed a significantly higher abundance of

Latilactobacillus, Lactococcus, and lower *Lactiplantibacillus* compared to all other vegetables (Figure 1B). Within-vegetable variation was assessed by calculating pairwise Bray-Curtis dissimilarity values (Figure 1D). These analyses confirmed that cucumber varied most across samples, while sauerkraut fermentations appeared most similar.

Despite these differential abundance profiles and differences in overall composition, a consistent pattern observed across most fermentations was that LAB, and especially *Leuconostoc*, were dominant across most fermentations, with its the composition and diversity varying depending on the vegetable substrate tested.

Salt reduction slows down LAB dominance and increases *Enterobacterales* abundance

While the vegetable substrate plays a key role in shaping microbial communities, environmental factors must also be considered in these man-made ecosystems. Among these, salt addition is one of the primary interventions used to steer microbial dynamics in fermented vegetables. To better understand its influence, we investigated how different salt concentrations influence active microbial community composition and the potential suppression of spoilage organisms and pathogens. We selected carrot juice fermentation as a model system, based on our prior experience with this ecosystem and its practical advantages in the laboratory. Its liquid nature, in particular, makes it easier to handle and sample under controlled conditions [12,24]. Carrot juice fermentations were set up with three salt concentrations, 2.5%, 1.25%, and 0% NaCl, to mimic standard conditions, lower salt concentration, and no salt conditions, respectively, Samples were collected over a 28-day period. As observed in the various vegetable fermentations, LAB were the most predominant members with low variation between different biological replicates. The microbial community initially consisted primarily of heterofermentative *Leuconostoc* and gradually shifted to a more homofermentative *Lactiplantibacillus*-dominated community, depending on the salt concentration (Figure 2A).

Alpha diversity was used as a proxy for fermentation consistency, with lower diversity indicating a more uniform LAB-dominated community (Figure 2B). Compared to 2.5% NaCl, significantly higher alpha diversity was observed up to day 14 in fermentations with 0% and 1.25% salt. By day 28, diversity in the 1.25% and 2.5% salt groups converged, suggesting that 1.25% NaCl fermentations progressed more slowly but eventually reached a similar *Lactiplantibacillus*-dominated state. In contrast, diversity remained higher in the no-salt group, representing lower LAB dominance.

Differential abundance analysis revealed that on day 0, fermentations with all salt concentrations displayed high levels of *Pseudomonas*, *Yersinia*, *Chimaeribacter*, and the unclassified *Enterobacterales* taxon BIGb0383, though at varying abundances. When 2.5% salt was added, *Leuconostoc* became dominant (>50% relative abundance) after 2 days, while under 1.25% and 0% salt conditions, this shift occurred after 7 days (Figure 2C). A similar delay was observed for *Lactiplantibacillus*, which dominated by day 14 in 2.5% salt, but only by day 28 in lower salt conditions. Notably, in the no-salt group, only 2 out of 3 fermentations still reached *Lactiplantibacillus* dominance by day 28. Low and no-salt fermentations also showed prolonged presence of potential spoilage taxa such as *Yersinia*, *Pseudomonas_E*, *Chimaeribacter*, and *BIGb0383*. *Weissella* was more abundant at nearly all timepoints in low-salt conditions, while *Lactococcus_A* increased later in fermentation. Taken together, these results indicate that reducing salt hampers LAB succession and allows spoilage-associated taxa to persist longer.

CO_2 addition under low salt conditions restores rapid LAB community domination

When lower salt concentrations are desired, alternative strategies are required to ensure LAB dominance. Therefore, we next investigated the potential of CO_2 as a selective intervention to support LAB-driven fermentation. Hereto, carrot juice fermentations were performed with or without prior CO_2 injection. Notably, CO_2-treated fermentations started a lower pH (6.08 ± 0.03 vs 5.43 ± 0.03; $p < 0.05$; Figure 3A), suggesting that CO_2 injection led to an initially more acidic environment. Despite this initial decrease, the overall pH trajectory during the fermentation was not altered in later stages. Interestingly, by day 2, pH values in CO_2-treated fermentations were slightly higher than in non-treated controls ($p < 0.05$), suggesting a small temporary buffering effect despite the initially lower pH. CO_2 injection significantly influenced early fermentation dynamics. Similar to the fermentations without CO2-injection, the communities were dominated by *Leuconostoc*, followed by the homofermentative *Lactiplantibacillus*. However, in the later stages, some samples with lower salt concentration contained *Lentilactobacillus* (Figure 3B). Alpha diversity remained significantly lower in CO_2-treated fermentations compared to controls until day 7, after which no differences were observed (Figure 3C).

Differential abundance analysis at the genus level showed that CO_2 injection led to a significant reduction in *Yersinia, Chimaeribacter*, and BIGb0383 on days 3 and 7, even in the absence of salt (Figure 3C-D). In parallel, *Leuconostoc* was significantly more abundant in CO_2-treated fermentations at these timepoints. Similar to the non-treated fermentation, *Weissella* is also increased in low-salt conditions that were treated with CO_2. Overall, CO_2 consistently promoted a LAB-dominated, low-Enterobacterales community structure.

These findings demonstrate that CO_2 injection at the start of fermentation can effectively steer microbial succession, suppress spoilage-associated taxa, and enhance LAB dominance, especially under low- or no-salt conditions. This highlights CO_2 as a promising tool to modulate fermentation ecosystems toward safer and more desirable outcomes.

Discussion

This study demonstrates how fermented vegetables can serve as tractable and engineerable model ecosystems for investigating microbial community assembly and succession. By systematically varying environmental parameters, including substrate type, salt concentration, and CO_2 levels, we observed reproducible patterns of microbial dominance, highlighting how ecological factors can be leveraged to steer microbial communities. These findings reinforce the value of fermented food systems as experimentally accessible platforms for testing ecological hypotheses in complex yet controllable environments.

Understanding bacterial community dynamics and competitive interactions is essential for optimizing the flavor, safety, and quality of fermented vegetables. While previous studies have typically focused on individual vegetable [12,16,19,47,48], we performed a standardized longitudinal analysis of the active community across 11 different vegetables. Specifically, the standardized handling was shown to be important as previous work documented that fermentation dynamics were influenced by handling and cutting types of fresh vegetables [49].

In our current study, we found that the specific vegetable substrate influenced overall bacterial composition most, while the region of plant (Carposphere, phyllosphere or rhizosphere) also influenced overall bacterial composition, but to a lesser extent. Most fermentations were consistently dominated by LAB, predominantly *Leuconostoc*. Remarkably, tomato and fennel fermentations were dominated by the uncharacterized Enterobacteriaceae taxon BIGB0383, which has also been detected in the *Caenorhabditis elegans* gut [50]. Given its close phylogenetic relationship to *Pseudescherichia*, an environmental microorganism which functionality remains ambiguous [51,52]. However, it is important to note that, due to the limitations of the current approach, taxonomic identification was only possible at the genus level. As such, we could not assess the presence of virulence factors or toxins, which would require strain-level resolution.

We then explored the importance of externally controllable factors such as salinity for steering vegetable fermentations. Reducing salt concentration clearly shifted the temporal microbial community dynamics. Lower salt concentrations were associated with higher alpha diversity compared to the reference condition of 2.5% NaCl, accompanied by the presence of less favorable *Enterobacterales* including the genera *Yersinia* and *Chimaeribacter,* as well as the non-*Enterobacterales* genus *Pseudomonas*. A slower microbial succession from heterofermentative *Leuconostoc* to homofermentative *Lactiplantibacillus*-dominated communities was also observed in lower salt concentrations. This trend partially aligned with findings in kimchi, who reported a lower abundance of homofermentative *Latilactobacillus* at lower salt concentrations, although this study

was limited to a single replicate[19]. Others have also observed elevated *Enterobacteriaceae* counts at lower salt concentrations in fermented cabbage [30], but they only measured colony-forming units of specific taxa of interest while our sequencing approach of the active fraction (RNA) allowed for a more comprehensive overview. Together, our findings clearly demonstrate that reducing salt concentrations not only increases the abundance of unwanted *Enterobacteriaceae* but also prolongs their presence, potentially leading to the accumulation of undesirable compounds such as biogenic amines [53].

We also investigated atmospheric conditions by focusing on steering CO_2 addition as an alternative strategy to modulate this ecosystem. Saturation of the fermentation matrix with CO_2 in carrot juice successfully induced a more rapid decline of *Enterobacterales* and a clear dominance of *Leuconostoc*. While an immediate pH reduction was observed following CO_2 introduction, acidification alone could not fully account for the suppression of *Enterobacterales*, as these taxa persisted at much lower pH in control fermentations with 2.5% NaCl. Prior studies have reported the antimicrobial effects of both high-pressure and dissolved CO_2[32,54–57], supporting the hypothesis that CO_2 directly contributed to the observed shifts in community structure. Our results demonstrated that CO_2 addition consistently decreased alpha diversity, suppressed the growth of potential spoilage organisms, and enhanced the predominance of beneficial *Lactobacillales* across different salt concentrations. Shifts within the LAB community were also observed, including an increase in *Weissella* at lower salt levels. However, a low but non-negligible *Yersinia* concentration was present in various conditions, including the no-salt CO_2-treated fermentation and the 2.5% salt without CO_2 injection. This indicates that a combinatorial approach could be beneficial to engineer this ecosystem to minimize the presence of undesirable taxa as early and effectively as possible. Additionally, the addition/flushing of CO_2 should be validated in various fermentation to reduce possible side effects like the bloater effect in cucumbers [58]. Overall, CO_2 addition emerges as a promising strategy to enhance fermentation safety while reducing salt content, opening opportunities for robust and healthier fermented vegetable products.

The results from our current study can be used to explore questions about the ecological mechanisms driving LAB succession in vegetable fermentations. While high salinity has long been considered a key selective pressure favoring LAB, our results suggest that other environmental factors such as the addition of CO_2 can be used modulate the microbial community assembly. Of note, heterofermentative LAB such as *Leuconostoc* also produce CO_2 as a metabolic byproduct, which may further also positively influence community dynamics during fermentation.

In summary, this study addressed three key knowledge gaps in the field of vegetable fermentation. First, it showed a systematic LAB dominance of the microbial community across a wide range of

vegetable types. Second, it demonstrated the essential role of salt on microbial community dominance and successions. Third, it revealed the potential of CO_2 addition to simultaneously restore natural microbial dynamics and enable food-safe salt reduction in vegetable fermentations. Given its promising impact, further research into the scalability and robustness of CO_2 injection in larger fermentation systems is warranted. More broadly than merely food fermentation applications, this study illustrates that targeted manipulation of environmental parameters can be used to steer microbial ecosystems.

Acknowledgements

The authors would like to acknowledge all colleagues from the Lab of Applied Microbiology and Biotechnology, with special thanks to Nele and Sam for their contributions. We also sincerely thank prof. Paul Cotter for his insightful feedback on this work. Finally, we acknowledge the third-year Bioscience Engineering bachelor students of 2021 and 2022 for their efforts in initiating the vegetable fermentation.

Funding

ERC starting grant Lacto-Be 852600 of SL supporting TE, TG, SW, and WVB. TE is partially funded by VLAIO (HBC.2022.1000). WVB, SW, and MV were funded by grants from Research Foundation – Flanders (FWO, grants 1224923N, 1S08523N, and 1SC2725N, respectively).

Conflict of interest

S.L. declares to be a voluntary academic board member of ISAPP (the International Scientific Association on Probiotics and Prebiotics, www.isappscience.org), cofounder of YUN and scientific advisor for Freya Biosciences. T.E. is partially funded through an industrial research VLAIO grant not related to this work. P.A.B. is an independent consultant for several companies in the food and pharma industry, bound by confidentiality agreements. The remaining authors have no conflicts of interest to declare. However, the companies were not involved in this paper.

Data availability

Amplicon sequencing data will be made available on ENA (PRJEB98055) upon publishing.

Figure legends

Figure 1: ***Active bacterial community structure of different vegetables fermented with 2 % salt added.*** Vegetable fermentations were ordered based on a hclust on the Maaslin2 effect sizes. *A: Overview of the relative abundances of the different vegetable fermentations on day 7 and day 14. The bar plot illustrates genus relative abundances in samples from day 7 and 14. B: Differential abundances of the top 11 most dominant taxa across all fermentations assessed with 8 different differential abundance metrics for each vegetable tested against the collective group. Color shows maaslin2 effect size. Number represent the total number of statistical tests that were significant with FDR corrected p-value <0.05. C: Alpha diversity for each vegetable. D: pairwise Bray-Curtis dissimilarity within vegetables. The color shows the direction of the effect size compared to all other vegetables, p-value <0.05 "*".*

Figure 2: ***Impact of salt concentration on carrot juice fermentation A****: Bar plot illustrating carrot juice fermentations with three replicates of three different salt conditions over time* ***B****: alpha diversity calculated with inverse Simpson over time compared to the samples with a reference salt concentration of 2.50%NaCl. Different shades of red represent the different salt (NaCl) concentrations used.* ***C****: A heatmap comparison of the differential abundances compared to the reference salt concentration of 2.50%NaCl of the 11 most abundant taxa over time, calculated with 8 different differential abundance tests, with effect size visualized by maaslin2. (p-values were corrected for false discovery rate FDR, "*"= p <0.05). Number depicts the number of significant differential abundance tests.*

Figure 3: ***Influence of CO_2 on microbial community dynamics in carrot juice fermentations.*** *Shades of red present the fermentations with CO_2 treatment (CO_2), while shades of blue represent the fermentations without CO2 injected.* ***A****: pH of carrot juice fermentations with various salt concentration and addition of CO_2 before the start of fermentation.* ***B****: Bar plot illustrating the microbial community after addition of CO_2 in the fermentation under different salt concentrations, for the control, see Figure 2A.* ***C****: Alpha diversity over time compared to the reference salt concentration of 2.50% NaCl.* ***D****: Differential abundance CO_2-injected fermentations compared to 2.5% NaCl without CO_2 determined with Maaslin2. ("*"=FDR corrected p<0.05).*

Reference list

1. Wolfe, B. E. & Dutton, R. J. Fermented foods as experimentally tractable microbial ecosystems. *Cell* 161, (2015).

2. Marsh, A. J., Hill, C., Ross, R. P. & Cotter, P. D. Fermented beverages with health-promoting potential: Past and future perspectives. *Trends Food Sci. Technol.* 38, (2014).

3. Marco, M. L. *et al.* Health benefits of fermented foods: microbiota and beyond. *Curr. Opin. Biotechnol.* 44, (2017).

4. Feng, R., Chen, L. & Chen, K. Fermentation trip: amazing microbes, amazing metabolisms. *Ann. Microbiol.* 68, (2018).

5. Mukherjee, A., Breselge, S., Dimidi, E., Marco, M. L. & Cotter, P. D. Fermented foods and gastrointestinal health: underlying mechanisms. *Nat. Rev. Gastroenterol. Hepatol.* (2023).

6. Wei, L., Van Beeck, W., Hanlon, M., DiCaprio, E. & Marco, M. L. Lacto-Fermented Fruits and Vegetables: Bioactive Components and Effects on Human Health. *Annu. Rev. Food Sci. Technol.* 16, (2025).

7. Marco, M. L. *et al.* The International Scientific Association for Probiotics and Prebiotics (ISAPP) consensus statement on fermented foods. *Nat. Rev. Gastroenterol. Hepatol.* 18, (2021).

8. Gänzle, M. G. The periodic table of fermented foods: limitations and opportunities. *Appl. Microbiol. Biotechnol.* 106, (2022).

9. Tamang, J. P., Watanabe, K. & Holzapfel, W. H. Review: Diversity of microorganisms in global fermented foods and beverages. *Front. Microbiol.* 7, (2016).

10. Wuyts, S., Van Beeck, W., Allonsius, C. N., van den Broek, M. F. & Lebeer, S. Applications of plant-based fermented foods and their microbes. *Current Opinion in Biotechnology* 61, (2020).

11. Gänzle, M. G. Lactic metabolism revisited: Metabolism of lactic acid bacteria in food fermentations and food spoilage. *Curr. Opin. Food Sci.* 2, (2015).

12. Wuyts, S. *et al.* Carrot juice fermentations as man-made microbial ecosystems dominated by lactic acid bacteria. *Appl. Environ. Microbiol.* 84, (2018).

13. Zabat, M. A., Sano, W. H., Wurster, J. I., Cabral, D. J. & Belenky, P. Microbial community analysis of sauerkraut fermentation reveals a stable and rapidly established community. *Foods* 7, (2018).

14. Zhang, S., Zhang, Y., Wu, L., Zhang, L. & Wang, S. Characterization of microbiota of naturally fermented sauerkraut by high-throughput sequencing. *Food Sci. Biotechnol.* 32, (2023).

15. Jung, M. J. *et al.* Role of combinated lactic acid bacteria in bacterial, viral, and metabolite dynamics during fermentation of vegetable food, kimchi. *Food Res. Int.* 157, (2022).

16. Song, H. S. *et al.* Effects of the main ingredients of the fermented food, kimchi, on bacterial composition and metabolite profile. *Food Res. Int.* 149, (2021).

17. Pérez-Díaz, I. M. *et al.* Reassessment of the succession of lactic acid bacteria in commercial cucumber fermentations and physiological and genomic features associated with their dominance. *Food Microbiol.* 63, (2017).

18. Van Beeck, W. Analysis of the food safety and microbial ecology of fermented carrot juice. *PhD Thesis* (2021).

19. Lee, M. A. *et al.* Influence of salinity on the microbial community composition and metabolite profile in Kimchi. *Fermentation* 7, (2021).

20. Yu, A. O., Leveau, J. H. J. & Marco, M. L. Abundance, diversity and plant-specific adaptations of plant-associated lactic acid bacteria. *Environ. Microbiol. Rep.* 12, (2020).

21. Leech, J. *et al.* Fermented-Food Metagenomics Reveals Substrate-Associated Differences in Taxonomy and Health-Associated and Antibiotic Resistance Determinants. *mSystems* 5, (2020).

22. Bokulich, N. A., Thorngate, J. H., Richardson, P. M. & Mills, D. A. Microbial biogeography of wine grapes is conditioned by cultivar, vintage, and climate. *Proc. Natl. Acad. Sci. U. S. A.* 111, (2014).

23. Di Cagno, R., Coda, R., De Angelis, M. & Gobbetti, M. Exploitation of vegetables and fruits through lactic acid fermentation. *Food Microbiol.* 33, (2013).

24. Van Beeck, W. *et al.* Robustness of fermented carrot juice against Listeria monocytogenes, Salmonella Typhimurium and Escherichia coli O157:H7. *Int. J. Food Microbiol.* 335, (2020).

25. Anekella, K. & Pérez-Díaz, I. M. Characterization of robust Lactobacillus plantarum and Lactobacillus pentosus starter cultures for environmentally friendly low-salt cucumber fermentations. *J. Food Sci.* 85, (2020).

26. Bautista-Gallego, J., Rantsiou, K., Garrido-Fernández, A., Cocolin, L. & Arroyo-López, F. N. Salt reduction in vegetable fermentation: Reality or desire? *J. Food Sci.* 78, (2013).

27. Hoppu, U. *et al.* Effect of salt reduction on consumer acceptance and sensory quality of food. *Foods* 6, (2017).

28. Cappuccio, F. P., Beer, M. & Strazzullo, P. Population dietary salt reduction and the risk of cardiovascular disease. A scientific statement from the European Salt Action Network. *Nutr. Metab. Cardiovasc. Dis.* 29, (2019).

29. Mente, A. *et al.* Associations of urinary sodium excretion with cardiovascular events in individuals with and without hypertension: a pooled analysis of data from four studies. *Lancet* 388, (2016).

30. Yang, X. *et al.* Effect of salt concentration on microbial communities, physicochemical properties and metabolite profile during spontaneous fermentation of Chinese northeast sauerkraut. *J. Appl. Microbiol.* 129, (2020).

31. Pérez-Díaz, I. M. *et al.* Modulation of the bacterial population in commercial cucumber fermentations by brining salt type. *J. Appl. Microbiol.* 128, (2020).

32. Devlieghere, F. & Debevere, J. Influence of Dissolved Carbon Dioxide on the Growth of Spoilage Bacteria. *Lwt* 33, (2000).

33. Zheng, J. *et al.* A taxonomic note on the genus Lactobacillus: Description of 23 novel genera, emended description of the genus Lactobacillus beijerinck 1901, and union of Lactobacillaceae and Leuconostocaceae. *Int. J. Syst. Evol. Microbiol.* 70, (2020).

34. Callahan, B. J. *et al.* DADA2: High-resolution sample inference from Illumina amplicon data. *Nat. Methods* 13, (2016).

35. Parks, D. H. *et al.* GTDB: An ongoing census of bacterial and archaeal diversity through a phylogenetically consistent, rank normalized and complete genome-based taxonomy. *Nucleic Acids Res.* 50, (2022).

36. Quast, C. *et al.* The SILVA ribosomal RNA gene database project: Improved data processing and

web-based tools. *Nucleic Acids Res.* 41, (2013).

37. Wittouck, S., Rillaer, T. Van, Smets, W. & Lebeer, S. Tidytacos: An R package for analyses on taxonomic composition of microbial communities. *J. Open Source Softw.* 10, (2025).

38. Wickham, H. *et al.* Welcome to the Tidyverse. *J. Open Source Softw.* 4, (2019).

39. Lebeer, S. *et al.* A citizen-science-enabled catalogue of the vaginal microbiome and associated factors. *Nat. Microbiol.* 8, (2023).

40. Nixon, M. P., Gloor, G. B. & Silverman, J. D. Beyond Normalization: Incorporating Scale Uncertainty in Microbiome and Gene Expression Analysis. *bioRxiv* (2024).

41. Lin, H. & Peddada, S. Das. Multigroup analysis of compositions of microbiomes with covariate adjustments and repeated measures. *Nat. Methods* 21, (2024).

42. Martin, B. D., Witten, D. & Willis, A. D. Modeling microbial abundances and dysbiosis with beta-binomial regression. *Ann. Appl. Stat.* 14, (2020).

43. Love, M. I., Huber, W. & Anders, S. Moderated estimation of fold change and dispersion for RNA-seq data with DESeq2. *Genome Biol.* 15, (2014).

44. Ritchie, M. E. *et al.* Limma powers differential expression analyses for RNA-sequencing and microarray studies. *Nucleic Acids Res.* 43, (2015).

45. Mallick, H. *et al.* Multivariable association discovery in population-scale meta-omics studies. *PLoS Comput. Biol.* 17, (2021).

46. Yang, L. & Chen, J. A comprehensive evaluation of microbial differential abundance analysis methods: current status and potential solutions. *Microbiome* 10, (2022).

47. Choi, I. K. *et al.* Novel Leuconostoc citreum starter culture system for the fermentation of kimchi, a fermented cabbage product. *Antonie van Leeuwenhoek, Int. J. Gen. Mol. Microbiol.* 84, (2003).

48. Diaz, M. *et al.* Comparison of the microbial composition of African fermented foods using amplicon sequencing. *Sci. Rep.* 9, (2019).

49. Valence, F. *et al.* The cutting type of vegetables influences the spontaneous fermentation rate. *Peer Community J.* 5, (2025).

50. Zhang, F. *et al.* Natural genetic variation drives microbiome selection in the Caenorhabditis elegans gut. *Curr. Biol.* 31, (2021).

51. Kim, I., Woo, H., Chhetri, G., Park, S. & Seo, T. A novel exopolysaccharide-producing bacterium, Pseudescherichia liriopis sp. nov. isolated from Liriope platyphylla, enhances the growth of Daucus carota subsp. sativus under drought and salinity stress. *Front. Plant Sci.* 15, (2024).

52. Brenner, D. J., McWhorter, A. C., Knutson, K. L. & Steigerwalt, A. G. Escherichia vulneris: A new species of Enterobacteriacea associated with human wounds. *J. Clin. Microbiol.* 15, (1982).

53. Gardini, F., Özogul, Y., Suzzi, G., Tabanelli, G. & Özogul, F. Technological factors affecting biogenic amine content in foods: A review. *Front. Microbiol.* 7, (2016).

54. King, J. S. & Mabbitt, L. A. Preservation of raw milk by the addition of carbon dioxide. *J. Dairy Res.* 49, (1982).

55. Amit, S. K., Uddin, M. M., Rahman, R., Islam, S. M. R. & Khan, M. S. A review on mechanisms and commercial aspects of food preservation and processing. *Agric. Food Secur.* 6, (2017).

56. Rowe, M. T. Effect of carbon dioxide on growth and extracellular enzyme production by Pseudomonas fluorescens B52. *Int. J. Food Microbiol.* 6, (1988).

57. Fraser, D. Bursting bacteria by release of gas pressure [8]. *Nature* 167, (1951).

58. Zhai, Y. & Pérez-Díaz, I. M. Identification of potential causative agents of the CO2-mediated bloater defect in low salt cucumber fermentation. *Int. J. Food Microbiol.* 344, (2021).

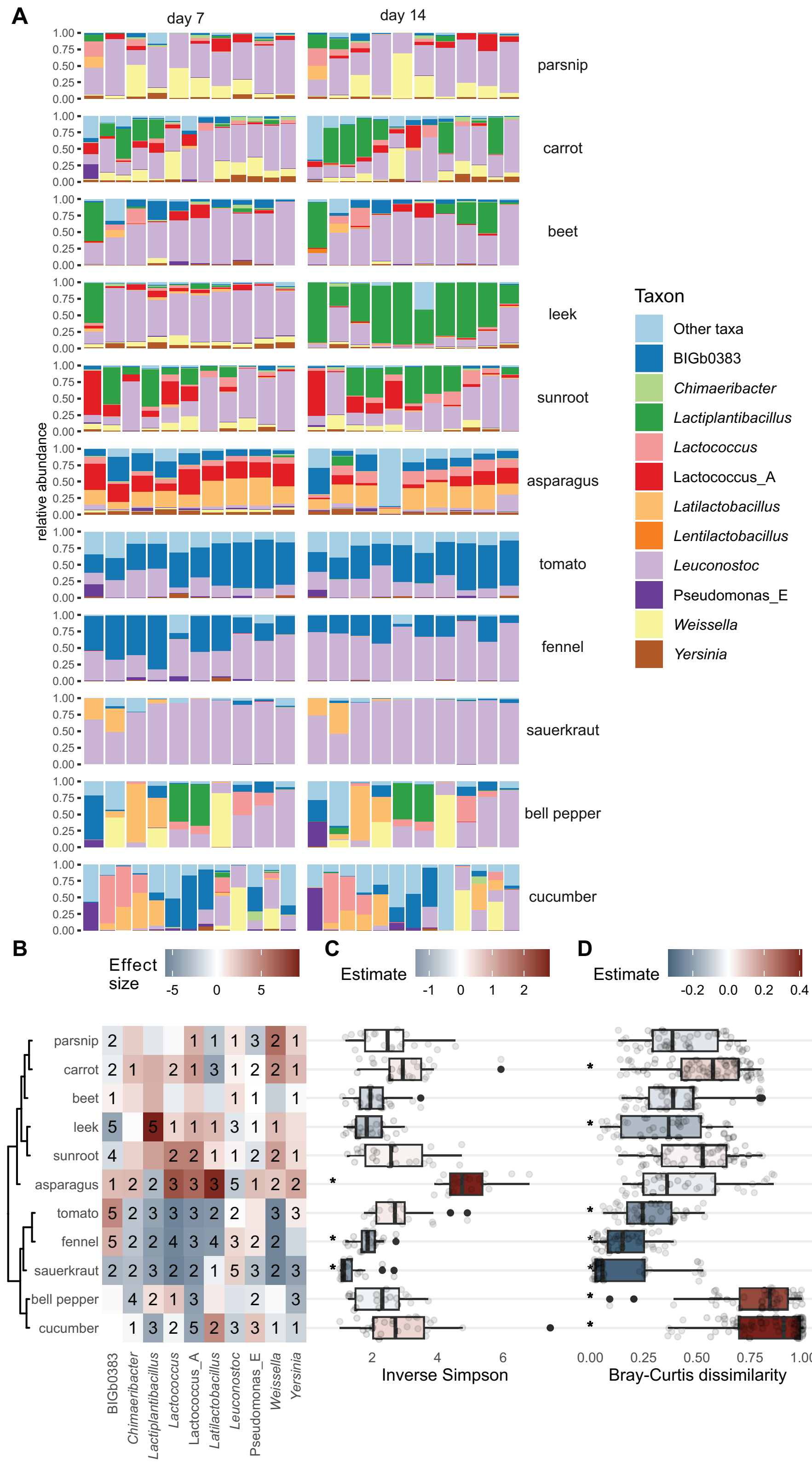
A
day 7
day 14
relative abundance
parsnip
carrot
beet
leek
sunroot
asparagus
tomato
fennel
sauerkraut
bell pepper
cucumber
Taxon
Other taxa
BIGb0383
Chimaeribacter
Lactiplantibacillus
Lactococcus
Lactococcus_A
Latilactobacillus
Lentilactobacillus
Leuconostoc
Pseudomonas_E
Weissella
Yersinia
B
Effect size
C
Estimate
D
Estimate
Inverse Simpson
Bray-Curtis dissimilarity

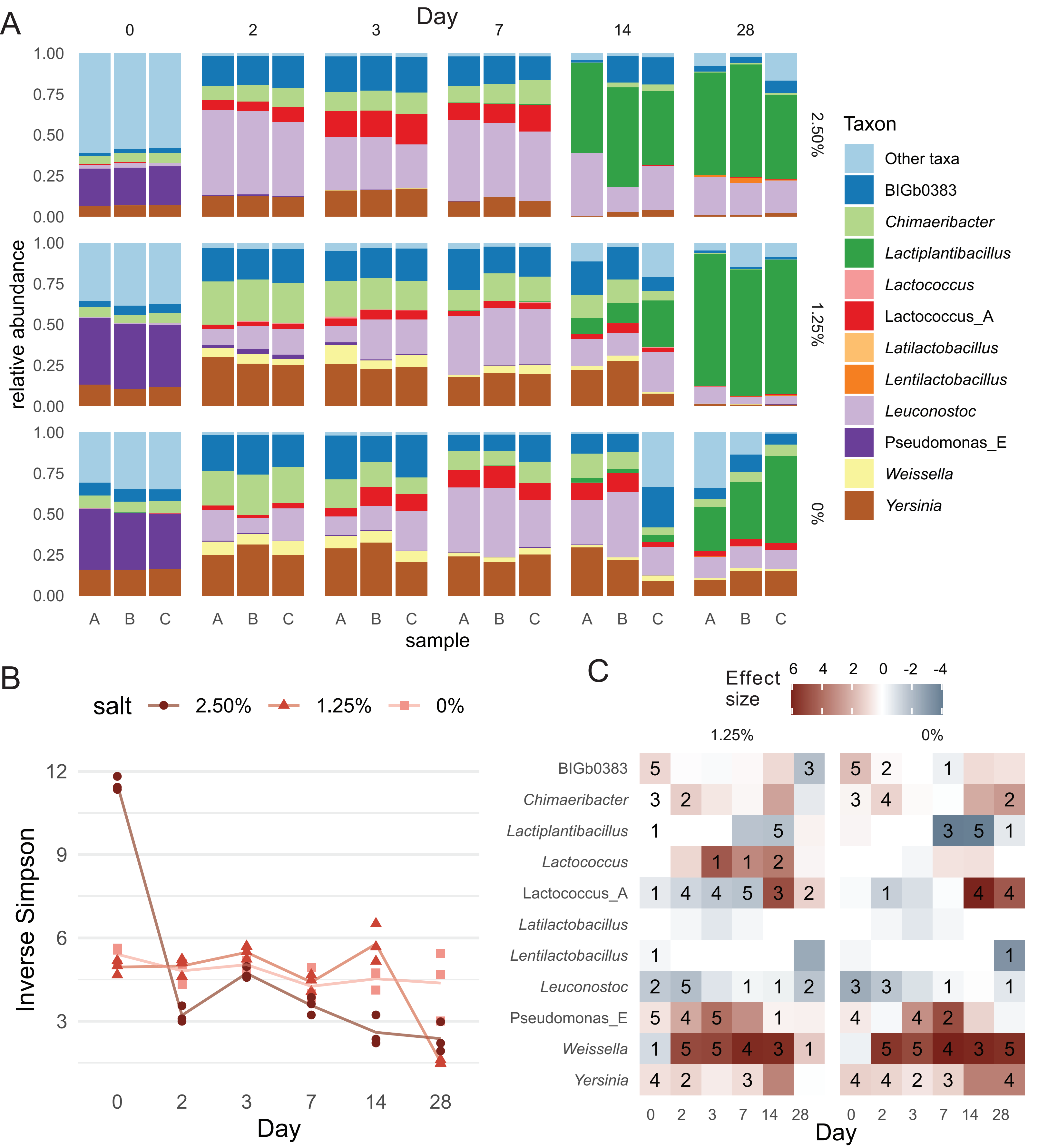
A
Day
0
2
3
7
14
28
relative abundance
1.00
0.75
0.50
0.25
0.00
2.50%
1.25%
0%
A B C
sample
Taxon
Other taxa
BIGb0383
Chimaeribacter
Lactiplantibacillus
Lactococcus
Lactococcus_A
Latilactobacillus
Lentilactobacillus
Leuconostoc
Pseudomonas_E
Weissella
Yersinia
B
salt
2.50%
1.25%
0%
Inverse Simpson
12
9
6
3
Day
C
Effect size
6 4 2 0 -2 -4
1.25%
0%
BIGb0383 5 3 5 2 1
Chimaeribacter 3 2 3 4 2
Lactiplantibacillus 1 5 3 5 1
Lactococcus 1 1 2
Lactococcus_A 1 4 4 5 3 2 1 4 4
Latilactobacillus
Lentilactobacillus 1 1
Leuconostoc 2 5 1 1 2 3 3 1 1
Pseudomonas_E 5 4 5 1 4 4 2
Weissella 1 5 5 4 3 1 5 5 4 3 5
Yersinia 4 2 3 4 4 2 3 4
0 2 3 7 14 28
Day

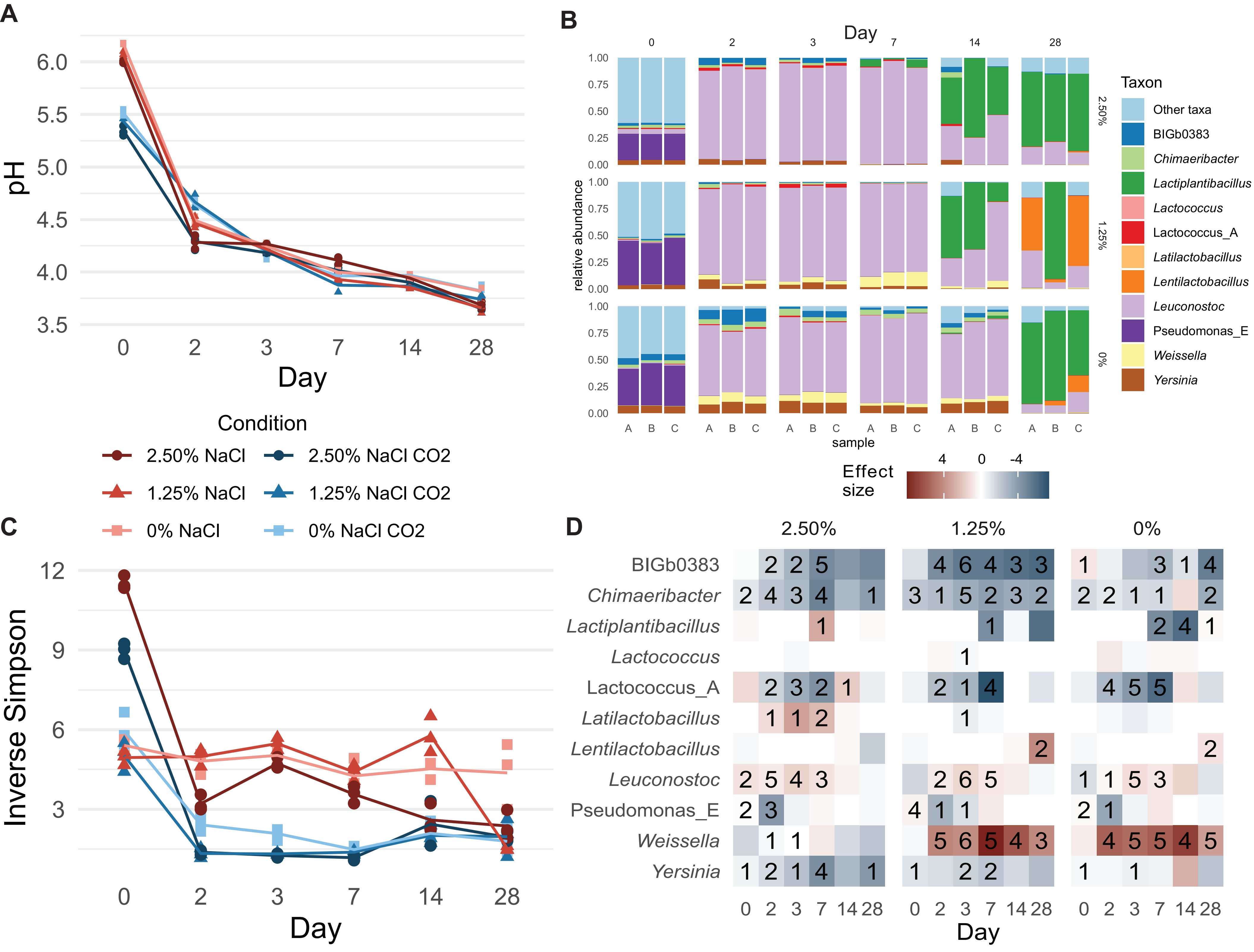
A
pH
Day
Condition
2.50% NaCl
2.50% NaCl CO2
1.25% NaCl
1.25% NaCl CO2
0% NaCl
0% NaCl CO2
B
Day
relative abundance
sample
2.50%
1.25%
0%
Taxon
Other taxa
BIGb0383
Chimaeribacter
Lactiplantibacillus
Lactococcus
Lactococcus_A
Latilactobacillus
Lentilactobacillus
Leuconostoc
Pseudomonas_E
Weissella
Yersinia
Effect size
C
Inverse Simpson
Day
D
2.50%
1.25%
0%
Day

Reviewer #1 (Comments for the Author):

This reviewer was quite happy with the initial submission and comments were adequately addressed. I remain with only few minor comments:

- It is somewhat unclear what the experimental unit is when experimenting with plants as source of fermentation microbes: This can be defined as the individual plant, or as a batch of plants that were grown on the same field (or purchased in the same (super)market at the same time). There is no right or wrong answer but: The response to reviews indicates that "plant material was sourced from different farms or stores" but the manuscript does not. If Table 1 can be modified to include the # of farms or stores in brackets after the number of replicates, this would clarify replication better.

> Answer: We thank the reviewer for this comment and have clarified this in the Methods section. Replication in this study was defined at the level of individual fermentation jars (the experimental unit throughout the analysis). Vegetables were obtained from varying numbers of farms or retail stores, but source was not used as a stratification factor for replication. Additionally, for smaller vegetables, multiple individual plants were used within a single fermentation, while for larger vegetables, multiple fermentations could be prepared from a single plant. Exact counts of plants per fermentation were not recorded. The text now reads:
>
> *Vegetables (Table 1) were sliced and fermented in 2.5% w/v NaCl solution, following a previously established protocol for carrot fermentation. For each vegetable, a minimum of nine independent fermentations replicates were prepared. Fermentations were conducted in Weck jars of 250 ml under controlled conditions, within a temperature-controlled room at 20°C. Depending on vegetable size, a single fermentation could contain material from multiple individual plants, while for larger vegetables, multiple fermentation could be prepared from a single plant. Samples (400 µL) were taken on days 7 and 14 from the same fermentation, snap-frozen in liquid nitrogen, and stored at -80°C for subsequent RNA extraction to determine the active community composition.*

line 385. This reviewer prefers simple answers and a reduced pH is a perfectly fine explanation to the faster dominance of Leuconostoc. To illustrate: a pH of 5.5 is nowhere close to inhibit even the most acid sensitive Enterobacteriaceae. To grow from initial numbers (10^4) to cell counts relevant in fermentations (10^7) about 10 generations. Leuconostoc requires additional generations as they occur in lower initial cell counts. Even a modest reduction of the generation time of Enterobacteriaceae (e.g. 30 to 45 min), which is likely achieved by acidification from pH 6.0 to 5.5, with an unaltered generation time of Leuconostoc could lead to initial dominance of leuconostoc instead of Enterobacteriaceae. The observation that a modest reduction of the pH shortcuts initial dominance of Enterobacteriaceae is known for olives (where vinegar is added to reduce the pH to 5 - 5.5) and for sponge dough fermentations (where baker's yeast consumes oxygen and reduces the pH to ~ 5.5 by CO2 production). This is not well documented in the scientific literature, though.

> **Answer**: We thank the reviewer for this hypothesis and agree that this mechanism is currently not well documented in the literature. While a study in olive fermentation indicates organic

acids (acetic and lactic acid) impact *Enterobacteriaceae* more than lactic acid bacteria, effects on relative growth rates are not explicitly addressed. We therefore included this hypothesis into the discussion section as an optional explanation. Specifically, we acknowledge that even a modest pH reduction can influence relative growth rates without full inhibition: The paragraph now states:

While an immediate pH reduction was observed following CO_2 introduction, acidification alone could not fully account for the suppression of Enterobacterales, as these taxa persisted at much lower pH in control fermentations with 2.5% NaCl. Nevertheless, even a slight pH reduction can influence relative growth rates without causing complete inhibition, reducing the initial dominance of Enterobacterales.

line 348. delete the lower case "f"

Answer: We have removed the lower case f.

Reviewer #2 (Public repository details (Required)):

I'm not sure the datasets were publicly deposited.

Answer: We thank the reviewer, and this data was still private on the ENA submission platform, now it has been made publicly available on PRJEB98055 (https://www.ebi.ac.uk/ena/browser/view/PRJEB98055)

Re: Spectrum03578-25R1 (From Diversity to Dominance: How Salt and CO_2 Shape LAB-dominated Ecosystems in Vegetable Fermentations)

Dear Prof. Sarah Lebeer:

Your manuscript has been accepted, and I am forwarding it to the ASM production staff for publication. Your paper will first be checked to make sure all elements meet the technical requirements. ASM staff will contact you if anything needs to be revised before copyediting and production can begin. Otherwise, you will be notified when your proofs are ready to be viewed.

Data Availability: ASM policy requires that data be available to the public upon online posting of the article, so please verify all links to sequence records, if present, and make sure that each number retrieves the full record of the data. If a new accession number is not linked or a link is broken, provide production staff with the correct URL for the record. If the accession numbers for new data are not publicly accessible before the expected online posting of the article, publication may be delayed; please contact ASM production staff immediately with the expected release date.

Publication Fees: For information on publication fees and which article types have charges, please visit our website. We have partnered with Copyright Clearance Center (CCC) to collect author charges. If fees apply to your paper, you will receive a message from no-reply@copyright.com with further instructions. For questions related to paying charges through RightsLink, please contact CCC at ASM_Support@copyright.com or toll free at +1-877-622-5543. CCC makes every attempt to respond to all emails within 24 hours.

ASM Membership: Corresponding authors may join or renew ASM membership to obtain discounts on publication fees. Need to upgrade your membership level? Please contact Customer Service at Service@asmusa.org.

Embargo Policy: A press release may be issued as soon as the manuscript is posted on the Spectrum Latest Articles webpage. The corresponding author will receive an email with the subject line "ASM Journals Author Services Notification" when the article is available online.

The ASM Journals program strives for constant improvement in our submission and publication process. Please tell us how we can improve your experience by taking this quick Author Survey.

Thank you for submitting your paper to Spectrum.

Sincerely,
Erik Hom
Editor
Microbiology Spectrum
